# Synthesis and biomedical applications of nanoceria, a redox active nanoparticle

**DOI:** 10.1186/s12951-019-0516-9

**Published:** 2019-07-10

**Authors:** Neelam Thakur, Prasenjit Manna, Joydeep Das

**Affiliations:** 1 School of Chemistry, Shoolini University of Biotechnology and Management Sciences, Bajhol, PO Sultanpur, Distt., Solan, 173229 HP India; 2 Biological Science and Technology Division, CSIR-North East Institute of Science and Technology, Jorhat, Assam 785006 India

**Keywords:** Nanoceria, Antibacterial activity, Antioxidant activity, Anti-cancer activity, Drug/gene delivery, Anti-diabetic effect

## Abstract

**Background:**

Nanoceria has recently received much attention, because of its widespread biomedical applications, including antibacterial, antioxidant and anticancer activity, drug/gene delivery systems, anti-diabetic property, and tissue engineering.

**Main body:**

Nanoceria exhibits excellent antibacterial activity against both Gram-positive and Gram-negative bacteria via the generation of reactive oxygen species (ROS). In healthy cells, it acts as an antioxidant by scavenging ROS (at physiological pH). Thus, it protects them, while in cancer cells (under low pH environment) it acts as pro-oxidant by generating ROS and kills them. Nanoceria has also been effectively used as a carrier for targeted drug and gene delivery in vitro and in vivo models. Besides, nanoceria can also act as an antidiabetic agent and confer protection towards diabetes-associated organ pathophysiology via decreasing the ROS level in diabetic subjects. Nanoceria also possesses excellent potential in the field of tissue engineering. In this review, firstly, we have discussed the different methods used for the synthesis of nanoceria as these are very important to control the size, shape and Ce^3+^/Ce^4+^ ratio of the particles upon which the physical, chemical, and biological properties depend. Secondly, we have extensively reviewed the different biomedical applications of nanoceria with probable mechanisms based on the literature reports.

**Conclusion:**

The outcome of this review will improve the understanding about the different synthetic procedures and biomedical applications of nanoceria, which should, in turn, lead to the design of novel clinical interventions associated with various health disorders.

**Graphical abstract:**

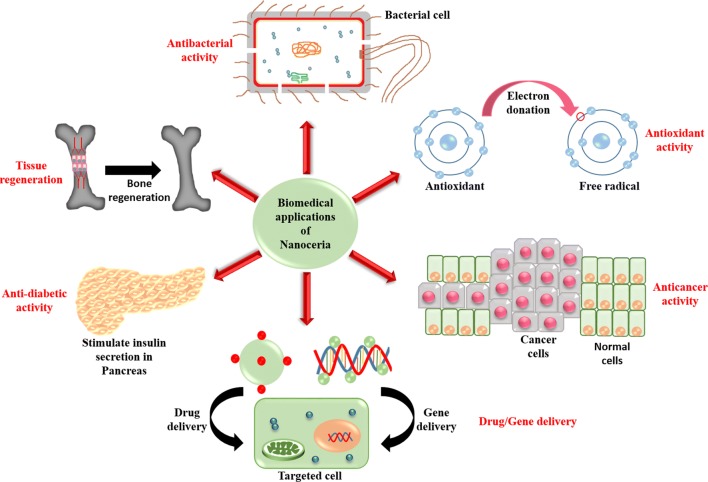

## Introduction

In recent years, there has been a significant advancement in the area of nanotechnology. Due to this, nanoparticles have received much attention in various fields such as in the environmental, industrial, and medical fields. Nanoparticles are the particles having size ranges between 1 and 100 nm. These particles possess unique properties such as small particle size, particle shape, higher surface area to volume ratio and have different electronic, magnetic, mechanical and optical properties compared to the bulk materials [1]. Nanoparticles should not be considered as a homogeneous group, as it includes a wide range of different materials having different physical and chemical properties. So, there are several types of nanoparticles such as: Fullerenes (C60, C70), carbon nanotubes (Multi-walled and Single-walled), various types of Metals (Au, Ag), Metal oxides (ZnO_2_, CeO_2_, TiO_2_), Quantum dots (CdSe, CdTe) [2], Liposomes, Dendrimers, Albumin-bound NPs, Polymeric NPs [3], Micelles [4], and Magnetic NPs [5]. There are two main approaches for the synthesis of nanoparticles: the top–down approach and the bottom-up approach. In the top–down approach, nanoparticles are prepared by decomposition of the larger molecule into smaller units which further form nanoparticles. This approach is also known as a destructive approach. Examples of this approach are grinding, physical vapor deposition, and chemical etching and various other decomposition techniques. While in the bottom-up approach, nanoparticles are prepared from elementary substances, and this approach is also known as building up approach. Examples of this approach are sedimentation, chemical vapor deposition, and various reduction techniques [6]. Nanoparticles show various applications in the biomedical field such as drug and gene delivery, bio-detection of pathogens, detection of proteins, tissue engineering, probing of DNA structure, separation, and purification of biological molecules, tumor destruction via heating and phagokinetic studies [7]. Nanoparticles also show good results in waste-water treatment because of their large surface area and thus helps in the purification of water [8].

Among various nanoparticles, metal oxide nanoparticles are essential and are widely used nowadays due to their unique properties and a large variety of applications in different fields. They are used in cosmetics, sunscreens, textiles, and self-cleaning coatings. These NPs are also widely used as water treatment agents, materials for solar batteries, and as automobile catalytic converters [9]. Some examples of metal oxide nanoparticles are zinc oxide, titanium oxide, iron oxide, cerium oxide nanoparticles, etc. All these metal oxide nanoparticles have their unique properties. Among these metal oxide nanoparticles, cerium oxide nanoparticles (Nanoceria) attract much interest due to its wide range of applications in different areas, especially in the biomedical field.

Cerium is the most abundant rare-earth element of the lanthanide series and found in earth’s crust. It can exist in both trivalent and tetravalent state. Cerium can also show stability in the tetravalent state, while other lanthanide elements are stable only in the trivalent state [10]. Cerium oxide or Nanoceria can be regarded as the most critical rare earth metal oxide with its unique ability to switch the oxidation states between (+ 3) and (+ 4) depending on the environment [11]. It can exist as both CeO_2_ and Ce_2_O_3_ in the bulk state [12] and shows catalytic activity due to the redox behavior of cerium [13]. It can adopt a fluorite crystalline lattice structure due to which it has a highly reactive surface area for the neutralization of free radicals [14]. With the decrease in the size of nanoceria, oxygen vacancies are formed in their lattice structure [15] and creating oxygen defects, thereby acting as a free radical scavenger in the physiological environment [16]. Nanoceria has been widely used in various areas such as, in fuel cells [17], optical devices [18], gas sensors [19], catalysis [20], ultraviolet absorbers [21], hydrogen storage materials [22], polishing materials [23], and biomedical fields [24]. However, there are only limited review papers describing the synthetic methodologies and biomedical applications of nanoceria [12, 25–27] and do not provide us complete information about their synthetic methodologies and biological applications. Therefore, in our present review article, we are aiming to combine all of the synthetic strategies along with their biomedical applications.

In this review, firstly, we have discussed various methods that are used to synthesize nanoceria. Among these methods, precipitation method is mostly used while green-synthesis method is advantageous and has given more consideration nowadays as it limits the toxicity of the solvents and reagents used. Then, we focused upon various biomedical applications of nanoceria, particularly the antibacterial activity, antioxidant activity, anticancer activity, drug/gene delivery applications, anti-diabetic activity, and tissue regeneration activity. These unique and extensive biomedical applications of nanoceria are advantageous for the treatment of various types of diseases. In the current review, we have critically analyzed and collected the desired information from previously published research and review papers to show the current state of nanoceria regarding its various biomedical applications. These unique properties of nanoceria will help the scientists and researchers to further explore its applications in the biomedical field.

## Synthesis of nanoceria

**Fig. 1:**
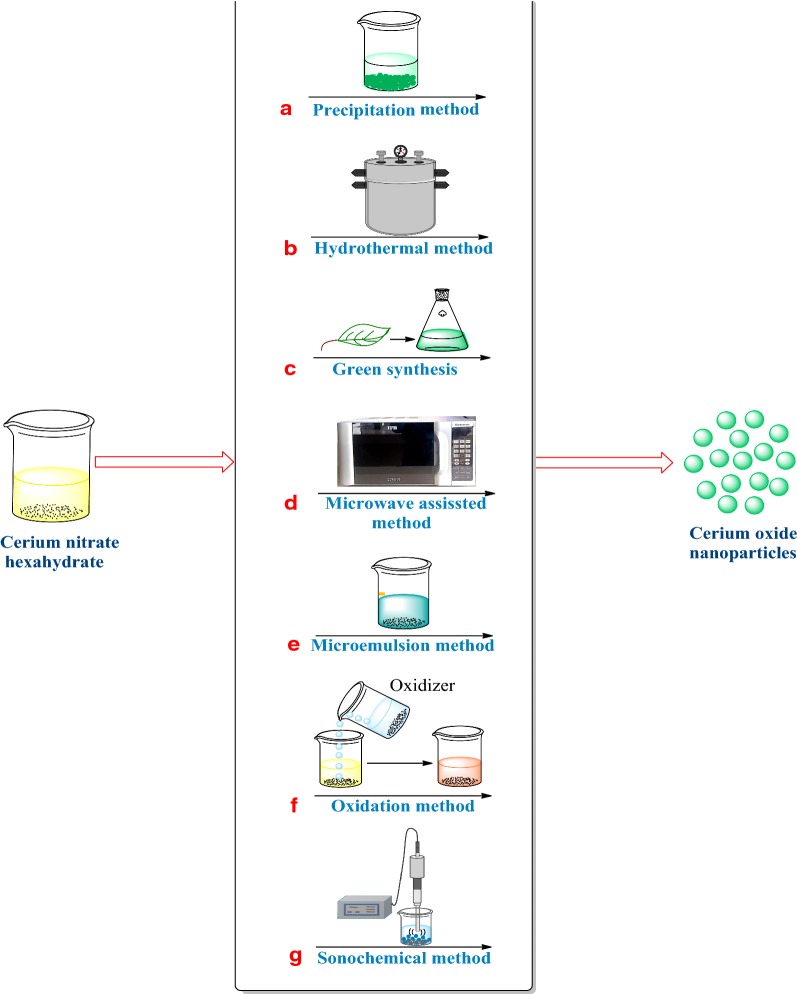
Different methods for the synthesis of nanoceria. **a** Precipitation method, **b** hydrothermal method, **c** green synthesis, **d** microwave-assisted method, **e** micro-emulsion method, **f** oxidation method and **g** sonochemical method

There are numerous methods for the synthesis of nanoceria. Those synthesis strategies are fundamental, as the physical and chemical properties of any nanoparticles depend upon them. So, here, we discuss the various methods which have been used to synthesize nanoceria (Fig. 1).

### Precipitation method

Precipitation method is the most convenient technique which has been used by several researchers for the synthesis of nanoceria, as shown in Table 1. In this method, nanoparticles can be synthesized either at room temperature or at a desired elevated temperature. In the year 1998, Terrible et al. [28] synthesized nanoceria using cerium chloride heptahydrate, cetyltrimethylammonium bromide (CTAB) surfactant and ammonia solution as precursors. The as-synthesized nanoceria (dimensions of 20-50A) were crystalline with high surface area and high thermal stability. Tsai et al. [29] reported the synthesis of nanoceria by homogeneous precipitation method using ammonium cerium nitrate and urea as precursors. The synthesized nanoceria had a cubic structure with the particle size of about 8 nm.

**Table 1:** Synthesis of nanoceria by precipitation method

S. no	Precursors	Medium	Method	References
1.	Cerium chloride heptahydrate, CTAB surfactant, and ammonia solution	Water	Precipitation	[28]
2.	Ammonium cerium nitrate and urea	Water	Homogeneous precipitation	[29]
3.	Cerium nitrate hexahydrate, ammonia solution, hydrogen peroxide, and hexamethylenetetramine	Water	Precipitation	[30]
4.	Hydrated Cerium acetate, sodium bis(2-ethylhexyl) sulfosuccinate, ammonia solution	Water and ethanol	Solution phase method	[43]
5.	Cerium nitrate hexahydrate and sodium hydroxide	Water	Precipitation	[31, 41]
6.	Cerium nitrate hexahydrate, ammonium hydroxide, acetone and tween 80	Acetone-water mixed solvent system	Surfactant-mediated precipitation method	[44]
7.	Cerium nitrate hexahydrate, dextran and ammonia solution	Water	Precipitation method	[35]
8.	Cerium nitrate, europium nitrate and ammonium hydroxide s	Water	Co precipitation	[36]
9.	Cerium nitrate hexahydrate, ammonia and ammonium bicarbonate	Water	Precipitation method	[37]
10.	Cerium nitrate hexahydrate, sodium hydroxide, and polyethylene glycol	Water	Chemical precipitation	[38]
11.	Cerium nitrate hexahydrate and aqueous ammonia	Water	Homogeneous precipitation	[39]
12.	Cerium chloride heptahydrate, CTAB surfactant, and ammonia solution	Water	Precipitation	[45]
13.	Cerium nitrate hexahydrate and aqueous ammonia	Water	Precipitation	[40]
14.	Cerium nitrate hexahydrate, EDTA and ammonium carbonate	Water	Chemical precipitation	[42]
15.	Cerium nitrate hexahydrate and potassium carbonate	Water	Co-precipitation method	[32]
16.	Cerium nitrate hexahydrate, polyacrylic acid, and ammonium hydroxide	Water	Precipitation method	[33]
17.	Cerium nitrate hexahydrate and ammonium hydroxide	Water	Precipitation method	[34]
18.	Cerium nitrate hexahydrate, xanthan gum, and ammonia solution	Water	Co-precipitation method	[46]

Most of the researchers used cerium nitrate hexahydrate as the common precursor for nanoceria synthesis in the presence of bases, such as aqueous ammonia, hexamethylenetetramine, NaOH, ammonium carbonate, and potassium carbonate [30–42].

Sun et al. [43] synthesized polycrystalline cerium oxide nanowires from hydrated cerium acetate, sodium bis (2-ethylhexyl) sulfosuccinate and ammonia solution as precursors. The diameter and length of as-prepared nanowires were in the range of 30–120 nm and 0.2–5 µm respectively.

Sujana et al. [44] reported a surfactant mediated precipitation method for the preparation of nanoceria by using cerium nitrate hexahydrate, ammonium hydroxide, acetone, and tween 80 surfactants. The synthesized nanoceria showed the average size of about 4.5 nm with a cubic fluorite structure and exhibited strong room-temperature photoluminescence as well as strong UV absorption. Later, Shelkar et al. [45] reported the synthesis of nanoceria by using cerium chloride hexahydrate, ammonium hydroxide, and CTAB surfactant.

Recently in 2018, Rahdar et al. [46] used a co-precipitation method to synthesize nanoceria from cerium nitrate hexahydrate, xanthan gum, and ammonia solution as precursors. The synthesized nanoceria was spherical having a size of about 20 nm.

All these data and information collected from previous literature showed that the precipitation method was mostly used by the researchers to synthesize nanoceria.

### Hydrothermal method

After precipitation method, this method is most commonly used by many researchers to synthesize nanoceria (Table 2). In the hydrothermal method, water is used as a solvent, and the chemical reaction takes place within autoclave in which the solution is heated under autogenous pressure. The first report of nanoceria synthesis by this technique is registered in 2002 by Masui et al. [47] who used cerium chloride hexahydrate, citric acid, and ammonia water as precursors. The nanoceria prepared under this condition was spherical with an average diameter of 5 nm. Citric acid acted as a protective agent to inhibit particle growth, which is further supported by the study of Renu et al. [48]. They prepared spherical nanoceria having a diameter ranging from 100 to 200 nm in the absence of citric acids.

**Table 2:** Synthesis of nanoceria by hydrothermal method

S. no	Precursors	Medium	Method	References
1.	Cerium chloride hexahydrate, citric acid, and ammonia water	Water	Hydrothermal crystallization	[47]
2.	Cerium nitrate and sodium hydroxide	Water	Hydrothermal method	[49]
3.	Cerium nitrate hexahydrate and sodium hydroxide	Water	Hydrothermal method	[50]
4.	Cerium nitrate hexahydrate and ammonium hydroxide	Water	Hydrothermal method	[53]
5.	Cerium nitrate hexahydrate and Polyvinyl pyrrolidone	Water	Surfactant-assisted hydrothermal method	[54]
6.	Ceric ammonium nitrate and ammonium carbonate	Water	Hydrothermal method	[55]
7.	Cerium chloride and ammonium hydroxide	Water	Hydrothermal method	[48]
8.	Cerium nitrate and sodium hydroxide	Water	Hydrothermal method	[51]
9.	Cerium nitrate hexahydrate and sodium hydroxide	Water	Hydrothermal method	[52]

Mai et al. [49] synthesized the same using cerium nitrate hexahydrate and sodium hydroxide. In their work, they synthesized nanoceria with three different morphologies; polyhedron having a size of 11.5 ± 1.8 nm, nanorods having a diameter of 9.6 ± 1.2 nm and nanocubes having a size of 36.1 ± 7.1 nm by changing the reaction temperature and base concentration. They observed a shape-dependent oxygen storage capacity of the synthesized nanoparticles; nanorods and nanocubes store the oxygen on their surface as well as in bulk, unlike nano polyhedron. After that, several other researchers prepared nanoceria by using the same precursors [50–52] too.

Patil et al. [53] synthesized positively charged nanoceria in the size range 8–10 nm from cerium nitrate hexahydrate and ammonium hydroxide. Zou et al. [54] reported the surfactant-assisted hydrothermal technique for the synthesis of nanoceria from cerium nitrate hexahydrate and polyvinyl pyrrolidone (PVP) as precursors. In this method, PVP was used as the surfactant. The nanoparticles synthesized were spherical having a diameter in the range of 5–10 nm and showed electrochemical properties.

Sutradhar et al. [55] used different precursors, such as ceric ammonium nitrate and ammonium carbonate to synthesize nanoceria. The as-prepared nanoceria was spherical having diameter 3–4 nm, possess high surface area, a large number of oxygen vacancies and showed perfect catalytic activity.

### Green/bio directed synthesis

Nowadays, researchers show more interest in Green synthesis approach for the preparation of nanoparticles as described in Table 3. The reason due to which this method has been given more consideration is related to the harmful effects associated with the chemical methods in which toxic chemicals have been used. The green synthesis approach is an easy, safer, reliable, efficient, and environment-friendly method which reduces the use or production of toxic substances. In this method, high temperature, pressure, and energy is not required. The main approaches involved in the synthesis of nanoceria by this method are nutrient-mediated synthesis, plant-mediated synthesis, fungus-mediated synthesis, and polymer-mediated synthesis [12].

**Table 3:** Synthesis of nanoceria by green/bio-directed synthesis

S. no	Precursors	Medium	Method	References
1.	Cerium(III) acetate hydrate and egg white	Water	Bio-directed synthesis	[56]
2.	Cerium chloride heptahydrate and *Acalypha indica* leaf extract	Water	Green approach	[59]
3.	Cerium nitrate hexahydrate, gum tragacanth from *Astragalus versus* and ammonia solution	Water	Gum mediated synthesis	[65]
4.	Cerium chloride heptahydrate and *Curvularia lunata* culture filtrate	Water	Mycosynthesis	[63]
5.	Cerium nitrate hexahydrate and aloe vera leaf extract	Water	Green method	[60]
6.	Cerium nitrate hexahydrate, starch, and ammonium hydroxide solution	Water	Green synthesis	[66]
7.	Cerium nitrate hexahydrate and honey	Water	Green synthesis	[67]
8.	*Gloriosa superba* leaf extract and cerium chloride salt	Water	Green synthesis	[58]
9.	Cerium nitrate hexahydrate and fresh egg white	Water	Bio-directed synthesis	[57]
10.	Cerium nitrate hexahydrate and *Hibiscus sabdariffa* flower extract	Water	Green synthesis	[62]
11.	Cerium nitrate hexahydrate and agarose	Water	Bio-organic polymer-based synthesis	[64]
12.	Cerium nitrate hexahydrate and *Olea europaea* leaf extract	Water	Green synthesis	[61]
13.	Cerium nitrate solution, pectin and ammonia solution	Water	Bio polymer mediated synthesis	[68]

Maensiri et al. [56] used nutrient-mediated synthesis approach for the preparation of nanoceria from cerium acetate hydrate and freshly extracted egg white as precursors. Nanoceria obtained by this method had a plate-like structure having an average diameter of 6–30 nm and showed excellent thermal, optical, and photoluminescence properties. Later, Kargar et al. [57] also used this approach for the synthesis of nanoceria from cerium nitrate hexahydrate and fresh egg white.

Arumugam et al. [58] used plant-mediated approach to synthesize nanoceria by using *Gloriosa superba* leaf extract and cerium chloride salt as precursors. The synthesized nanoceria was spherical with a particle size of 5 nm and showed antibacterial properties. Other researchers also reported the synthesis of nanoceria by this approach using different plant extracts such as *Acalypha indica* leaf extract [59], aloe vera leaf extract [60], *Olea europaea* leaf extract [61] and *Hibiscus sabdariffa* flower extract [62].

Fungus-mediated approach (Mycosynthesis) is also used by Munusamy et al. [63] to synthesize nanoceria from cerium chloride heptahydrate and *Curvularia lunata* culture filtrate. The nanoparticles synthesized by this method were tiny in size (5–20 nm) having a spherical shape and possessed antibacterial activity.

Polymer-mediated synthesis approach has also been taken by some researchers to synthesize nanoceria [64–67]. For example, Patil et al. [68] used cerium nitrate, pectin, and ammonia solution as precursors to synthesize nanoceria. The synthesized nanoparticles were spherical having an average particle size of ≤ 40 nm. This nanoceria showed antioxidant and antibacterial properties and was proved non-toxic towards the living tissues.

### Oxidation method

Oxidation method is a direct and straightforward method in which any suitable oxidizing agent (oxidizer) is used to synthesize nanoparticles. The preparation of nanoceria using this method has been adopted by several researchers, as shown in Table 4. Lee et al. [69] synthesized spherical nanoceria in water by simple oxidation of cerium ions using ammonium hydroxide as mineralizer and hydrogen peroxide as an oxidizer. In their work, they reported that the size of nanoceria was decreased with increase in the concentration of the oxidizer. Karakoti et al. [11] reported the synthesis of nanoceria in different biocompatible media such as water, polyethylene glycol, glucose, and dextran solution by using cerium nitrate hexahydrate as precursor while hydrogen peroxide and ammonia as an oxidizer in acidic and basic environment respectively. In comparison with the acidic medium, the nanoparticles synthesized in the primary medium showed less agglomeration. However, in both the conditions the size of synthesized nanoceria remained 3–5 nm. Some other precursors have also been used by different researchers to synthesize CNPs [30, 35, 70–79].

**Table 4:** Synthesis of nanoceria by oxidation method

S. no	Precursors	Medium	Method	References
1.	Cerium nitrate hexahydrate, hydrogen peroxide, and ammonia solution	Water	Hydrothermal oxidation method	[69]
2.	Cerium nitrate hexahydrate, ammonia solution, and hydrogen peroxide	Water	Oxidation method	[30]
3.	Cerium sulfate, sodium hydroxide, and hydrogen peroxide	Water	Sonochemical oxidation	[72]
4.	Cerium nitrate hexahydrate and hydrogen peroxide	Water	Oxidation	[11, 73–75]
5.	Cerium nitrate, polyethylene glycol, and hydrogen peroxide	20% polyethylene glycol solution	Oxidation	[76]
6.	Cerium nitrate hexahydrate, dextran and ammonium hydroxide	Water	Oxidation	[35, 77, 78]
7.	Cerium nitrate hexahydrate, polyacrylic acid, and ammonium hydroxide	Water	Oxidation	[78]
8.	Cerium (III) acetate hydrate and hydrogen peroxide	Water	Precipitation method	[79]
9.	Cerium nitrate hexahydrate and hydrogen peroxide	Polyethylene glycol	Oxidation	[70]
10.	Cerium nitrate hexahydrate, hydrogen peroxide, and ammonium hydroxide	Dextran	Oxidation	[71]

### Sonochemical method

In the sonochemical method, the reaction is done via the application of high-intensity ultrasound wave which forms acoustic cavitation, i.e., formation, growth and collapse of bubbles, resulting in the creation of very high local temperature and pressure that initiate a chemical reaction. These reactions are swift, and no external activation energy and pressure are applied [80]. Several researchers used this method to synthesize CNPs, as mentioned in Table 5.

**Table 5:** Synthesis of nanoceria by sonochemical method

S. no	Precursors	Medium	Method	References
1.	Cerium nitrate and azodicarbonamide	Water	Sonochemical synthesis	[81]
2.	Ammonium cerium nitrate, hexamethylenetetramine, and polyethylene glycol	Water	Sonochemical method	[82]
3.	Cerium nitrate hexahydrate, tetra ethylene glycol, and ammonium hydroxide	Water	Sonochemical method	[83]
4.	Cerium nitrate hexahydrate, CTAB surfactant, and ammonia solution	Water	Ultrasonication	[84]
5.	Cerium nitrate hexahydrate and aqueous ammonia solution	Water	Sonochemical hydrolysis method	[85]

The first sonochemical synthesis of nanoceria was reported in 2002 by Yin et al. [81] and Wang et al. [82]. Yin et al. [81] synthesized nanoceria using cerium nitrate and azodicarbonamide as precursors, while ethylenediamine or tetraalkylammonium hydroxide as additives. Those additives showed a potent effect on the size of nanoparticles. They observed that the addition of additives reduced the particle size, whereas, in the absence of additives, agglomerated nanoceria was obtained. On the other hand, Wang et al. [82] used ammonium cerium nitrate and hexamethylenetetramine as precursors for nanoceria synthesis.

Dutta et al. [83] synthesized flower-like nanoceria from cerium nitrate hexahydrate, tetraethylene glycol, and ammonium hydroxide. Later, several other researchers also synthesized nanoceria by a sonochemical method using cerium nitrate hexahydrate [84, 85].

### Microwave assisted method

The microwave-assisted method has been considered as one of the most sustainable methods for the synthesis of nanoceria. It is more efficient than any other conventional heating method because the microwaves couple directly with the solvent and reactant molecules present in the reaction mixture due to which the energy transfer takes place quickly leading to a rapid rise in the temperature of the system [86]. In this method, the reaction is completed within a short time, and spotless products are obtained with a higher yield [87]. Several researchers used this method for the preparation of nanoceria, as described in Table 6.

**Table 6:** Synthesis of nanoceria by microwave-assisted method

S. no	Precursors	Medium	Method	References
1.	Ammonium cerium nitrate, hexamethylenetetramine, and polyethylene glycol	Water	Microwave-assisted heating	[82]
2.	Ammonium cerium nitrate and sodium hydroxide	Water	Microwave-assisted hydrothermal method	[88]
3.	Cerium nitrate hexahydrate, ethylene glycol, oleic acid, and tert-butylamine	Water	Microwave-assisted method	[89]
4.	Cerium nitrate hexahydrate, propylene glycol, and ammonia	Water	Microwave technique	[90]
5.	Ceric ammonium nitrate and sodium hydroxide	Water	Microwave-mediated synthesis	[91]

Wang et al. [82] first synthesized nanoceria by microwave-assisted heating methods from ammonium cerium nitrate, hexamethylenetetramine and polyethylene glycol as precursors. A microwave oven with 650 W power was used in this method. The as-prepared nanoceria was spherical having an average diameter in the range of 2–3 nm.

Later, Gao et al. [88] synthesized cerium oxide nanoparticles and nanorods by a microwave-assisted hydrothermal method from ammonium cerium nitrate and using different base additives such as sodium hydroxide, ammonia water, urea, ethylene diamine, and formamide. The nanoceria prepared with different base additives possessed particle-like morphology but have different particle size. However, as prepared nanoparticles showed different morphologies when the cerium source was changed to cerium chloride and using a different quantity of ammonia water. Some other researchers also synthesized nanoceria by this method [89–91].

### Combustion method

Combustion method is also known as self-propagating high-temperature synthesis (SHS), which is a simple, rapid, and effective technique to prepare various nanoparticles. On the basis of the physical nature of initial reaction medium used, this method is further classified as, condensed phase combustion or Conventional SHS (in which the initial reaction medium is in solid state), solution–combustion synthesis (in which the initial reactants are in aqueous state) and gas phase combustion (in which nanoparticles are synthesized in flame) [92]. Synthesis of nanoceria by this method has been summarized in Table 7.

**Table 7:** Synthesis of nanoceria by combustion method

S. no	Precursors	Medium	Method	References
1.	Cerium nitrate hexahydrate, europium nitrate, and urea	Water	Solution combustion synthesis	[93]
2.	Cerium nitrate, rare earth nitrate, and anhydrous citric acid	Water	Combustion method	[94]
3.	Cerium nitrate hexahydrate, Sm(NO_3_)_3_, urea and PVA	Water	Combustion method	[95]
4.	Ceric ammonium nitrate and EDTA disodium salt	Water	Solution combustion method	[96]

Shi et al. [93] reported the synthesis of europium-doped nanoceria by solution combustion synthesis approach for the first time. They used cerium nitrate hexahydrate, europium nitrate, and urea as precursors, and the as-prepared nanoceria had an average size of 60 nm with photoluminescence and X-ray luminescence properties.

Later, Jamshidijam et al. [94] used combustion method to synthesize rare nanocrystalline earth-doped ceria nano-powders. They used a mixture of rare earth (RE) nitrates containing cerium and anhydrous citric acid. The size of the synthesized nanoceria was found to be 17–19.5 nm by using Scherrer’s formula. In the same year, Wu et al. [95] also synthesized nanoceria by a combustion method, using cerium nitrate hexahydrate, Sm(NO_3_)_3_, urea, and PVA as raw materials.

Ravishankar et al. [96] prepared nanoceria from ceric ammonium nitrate and EDTA disodium salt as precursors. The synthesized nanoceria was spherical with the average size of 42 nm and exhibited photocatalytic as well as antibacterial activity.

### Micro-emulsion method

Micro-emulsions are colloidal solutions having two immiscible solvents (water and oil), a surfactant and a co-surfactant. They can be prepared rapidly by stirring the above-mentioned components, and the formed microemulsions are thermodynamically stable solutions [97]. Microemulsions are of different types such as oil-in-water (O/W) microemulsions, water-in-oil (W/O) microemulsions, bicontinuous microemulsions, and supercritical CO_2_ microemulsions [98]. Micro-emulsion method has been adopted by several researchers for the synthesis of nanoceria as illustrated in Table 8.

**Table 8:** Synthesis of nanoceria by a microemulsion method

S. no	Precursors	Medium	Method	References
1.	Cerium nitrate hexahydrate, ammonium hydroxide and bis (2-ethylhexyl)sulphosuccinate	Toluene and water	Microemulsion method	[99, 100]
2.	Cerium nitrate hexahydrate sodium hydroxide and cetyl trimethyl ammonium bromide	Water and *n*-octane	Reverse micellar method or water in oil microemulsion	[101]
3.	Cerium nitrate hexahydrate, ammonium hydroxide, polyvinylpyrrolidone and butanol	Water and *n*-octane	Microemulsion method	[102]
4.	Cerium(III) 2-ethylhexanoate, ammonia, and hexaethylene glycol isodecyl ether	Water and hexane	Oil in water microemulsion	[103]

Patil et al. [99] and Das et al. [100] used this method to obtain nanoceria using cerium nitrate hexahydrate and ammonium hydroxide as precursors, bis (2-ethylhexyl) sulphosuccinate as a surfactant, and toluene and water as solvents. The formed nanoceria was monodispersed, having a size in the range of 5 nm with a spherical shape.

Sathyamurthy et al. [101] reported the synthesis of nanoceria by reverse micellar method or water in oil microemulsion method. They used the same precursor molecule, but different surfactant, such as cetyltrimethylammonium bromide and 1-butanol as a co-surfactant. Water and *n*-octane were used as aqueous and oil phase, respectively. The as-synthesized nanoceria had a polyhedral shape with the average size of about 3.7 nm and was found to exhibit room temperature photoluminescence and strong UV absorption.

Huang et al. [102] reported nanoceria synthesis via a similar method, where the same precursor molecule and solvent systems are used, but with a different surfactant, polyvinyl pyrrolidone (PVP). They synthesized nanoceria with different morphologies by controlling the pH of the solution. As the pH value increased from 5 to 8 to 11, the morphology of nanoceria transferred from granular to spherical and then to rod-like.

Tiseanu et al. [103], synthesized nanoceria via oil-in-water microemulsion method. They synthesized pure and europium doped nanoceria from Cerium (III) 2-ethyl hexanoate and europium (III) 2-ethyl hexanoate as precursors, hexaethylene glycol isodecyl ether as a surfactant, hexane solution as oil phase and water. The obtained nanoparticles possessed a very high surface area of ~ 250 m^2^/g having a size of about 3 nm.

### Sol–gel method

This method involves a few steps, such as hydrolysis, condensation, and drying, to form a final nanoparticle. Depending upon the type of solvent used, this method is further classified into two classes—aqueous sol–gel method (water is used as solvent) and non-aqueous sol–gel method (organic solvent is used). Nature of metal precursor and solvent plays a significant role in this method to synthesize metal oxide nanoparticles [104]. The sol–gel method is a considerable approach to synthesize nanoceria, as depicted in Table 9.

**Table 9:** Synthesis of nanoceria by Sol–gel method

S. no	Precursors	Medium	Method	References
1.	Cerium nitrate hexahydrate and oleic acid	Tri-*n*-octylamine, oleyamine and diphenyl ether	Nonhydrolytic sol–gel method	[105]
2.	Cerium nitrate hexahydrate and europium nitrate pentahydrate and citric acid monohydrate	Water and polyethylene glycol	Sol–gel method	[106]
3.	Cerium chloride heptahydrate and aqueous ammonia solution	Water and methanol	Sol–gel method	[107]

Non-hydrolytic sol–gel method has been used by Yu et al. [105], to synthesize nanoceria from cerium nitrate hexahydrate and diphenyl ether in oleylamine. The CNPs thus obtained were spherical having 3.5 nm in size. They also reported that anisotropic wire and tadpole-shaped nanoceria were obtained if oleic acid (cosurfactant) was added to the solution in addition to oleylamine.

Li et al. [106] prepared undoped and europium doped nanoceria by using the sol–gel method. They used cerium and europium nitrate as the starting materials, and water and polyethylene glycol as solvents. They reported the photoluminescence properties of the europium-doped nanoceria at different europium doping concentrations and different temperatures. In 2011, Gnanam et al. [107] also prepared CNPs from cerium chloride heptahydrate and aqueous ammonia solution as precursors, water, and methanol as solvents.

### Other methods

Besides the above discussed conventional methods, some researchers have also reported other methods for the preparation of nanoceria, as shown in Table 10. Some of these methods are:

**Table 10:** Synthesis of nanoceria by other methods

S. no	Precursors	Medium	Method	References
1.	Cerium acetate hydrate, oleyamine, oleic acid, and NaOH	Hexadecane	Nonhydrolytic solvent method	[108]
2.	Cerium nitrate hexahydrate and ethylene diamine	Ethylenediamine and water	Direct room temperature and solvothermal method	[109]
3.	Cerium nitrate hexahydrate and 6-aminohexanoic acid (AHA)	Water	Aqueous phase synthesis	[110]
4.	Cerium carbonate hydrate, and Molten KOH–NaOH mixture	Nil	Partial oxidation method	[111]
5.	Cerium nitrate	Water	Solution plasma process	[114]

- *Non-hydrolytic solvent method* Wang et al. [108] used this method for the preparation of nanoceria using cerium acetate hydrate, oleyamine, oleic acid, and NaOH as precursors in hexadecane medium. The as-prepared nanoceria had an average size of 3.5 nm with a cubic structure.
- *Direct room temperature and solvothermal method* Kar et al. [109] synthesized ultra-small cerium oxide nanoparticles having size 2.5 ± 0.2 nm from cerium nitrate hexahydrate and ethylene diamine by this method.
- *Aqueous phase synthesis* Yu et al. [110] reported the synthesis of ultrathin cerium oxide nanosheets by this method, using cerium nitrate hexahydrate and 6-aminohexanoic acid (AHA) as starting materials.
- *Partial oxidation method* This method was used to synthesize nanoceria by Lan et al. [111] using cerium carbonate hydrate in a molten KOH–NaOH mixture. They observed that with the increase in the synthesis temperature, the size of the synthesized nanoceria was also increased.
- *Solution plasma process* The solution plasma process is a new, fast, and efficient method for the preparation of nanoparticles. This is an electrical discharge process, which takes place in a wet environment, generally at room temperature and an atmospheric non-equilibrium plasma is produced [112]. The advantage of this method is that it can be carried out at ambient temperature and pressure without the addition of any harmful chemicals [113]. Davoodbasha et al. [114] reported the synthesis of nanoceria by discharging plasma in a cerium nitrate solution. The synthesized nanoceria was spherical and possessed an average size of 7.0 ± 0.2 nm and 5.0 ± 0.2 when the sample had been discharged with plasma for 15 min and 25 min respectively. The obtained nanoceria showed excellent antioxidant properties.

### Functionalization and loading of nanoceria with different ligands or drugs

Nanoceria has been functionalized with various ligands and loaded with drugs for targeted and effective drug delivery into cancer cells. Several researchers have used different functionalization or loading methods for different ligands and drugs. Patil et al. [115] functionalized nanoceria with carboxybenzenesulfonamide (a human carbonic anhydrase inhibitor) and carboxyfluorescein (a fluorophore) molecules via simple condensation reactions using epichlorohydrin as a linker molecule.

In 2017, Das et al. [116] loaded doxorubicin (a chemotherapeutic drug) on nanoceria by electrostatic interactions between negatively charged nanoceria and positively charged doxorubicin at pH 7.0.

Later in 2017, Sulthana et al. [33] reported the functionalization of nanoceria with polyacrylic acid (PAA) by simple alkaline precipitation of cerium ions in the presence of PAA. PAA coated nanoceria were further conjugated with propergylamine via amide bond formation, and the as-synthesized propergylated nanoceria was further conjugated with folic acid using “Click” chemistry. Finally, two drugs, namely ganetespib (an Hsp 90 inhibitor) and doxorubicin, were loaded onto the nanoceria via solvent diffusion method.

Kalashnikova et al. [34] prepared dextran functionalized nanoceria by simple alkaline precipitation of cerium ions in the presence of dextran. The as-prepared nanoceria was then further loaded with curcumin via metal–ligand chelation.

Zhang et al. [52] first prepared porous cerium oxide nanorods and then loaded doxorubicin inside the pores. After that, they coated the drug-loaded pores by a dithio-polydopamine layer by self-polymerization. Finally, the amine functionalized lactoses were conjugated on the polydopamine surface via a Michael addition reaction.

## Biomedical applications of nanoceria

Nanoceria has emerged as one of the vital metal oxide nanoparticles due to its wide range of applications in different fields, particularly in biomedicine. Here we have discussed its various biomedical applications.

### Antibacterial activity

**Fig. 2:**
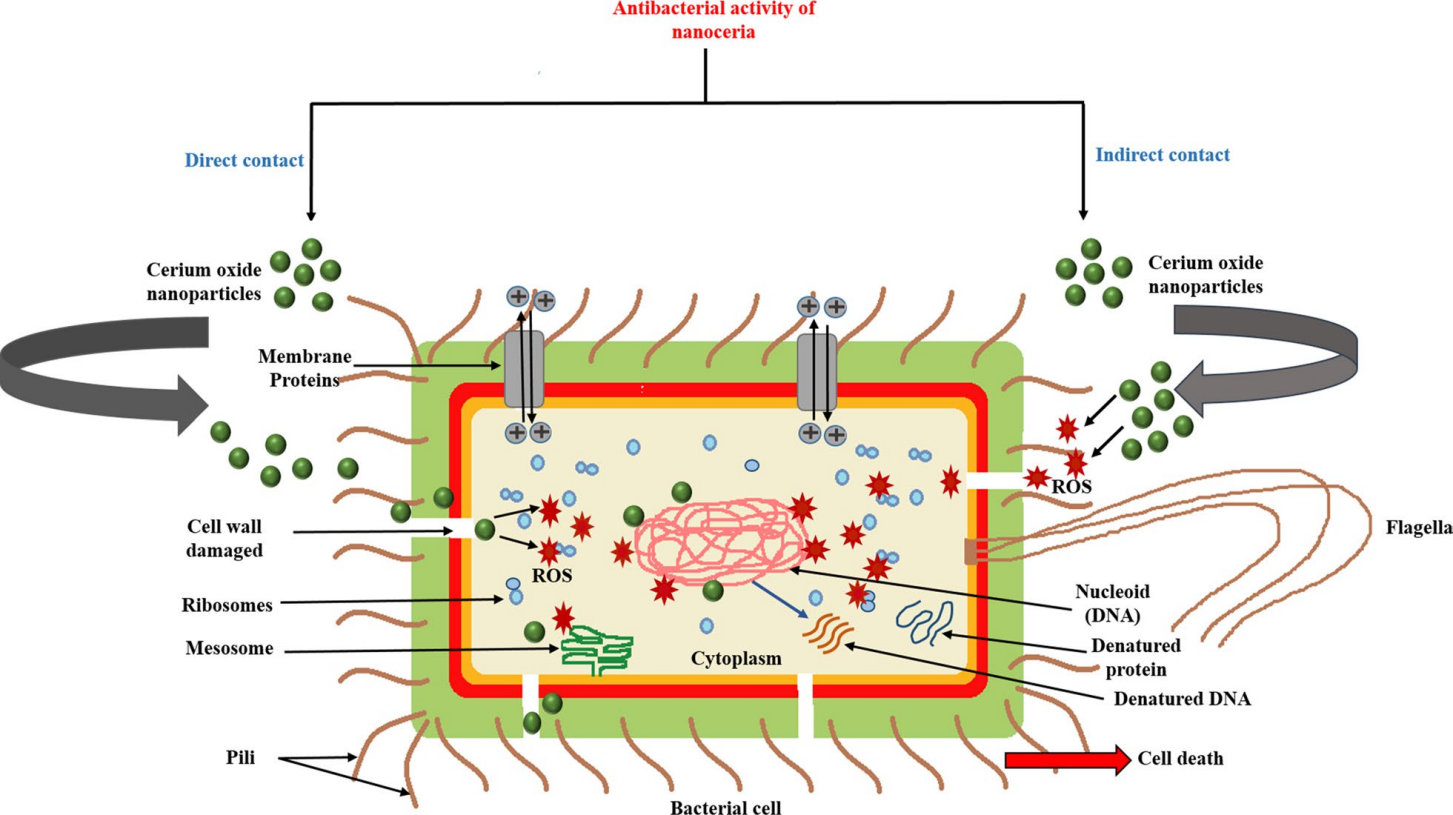
Mechanism of antibacterial activity of nanoceria. **a** Direct contact, showing the direct interaction of Cerium oxide nanoparticles (CNPs) with bacterial cell wall that damage the cell wall and gets penetrated inside the cell and generates reactive oxygen species (ROS) which affects DNA, ribosomes, and proteins. **b** Indirect contact, showing the interaction of CNPs with the bacterial environment outside the cell and generates ROS that further enters into the cell by damaging the cell wall and affects DNA, ribosomes, and proteins. Both mechanisms ultimately lead to cell death

Nanoceria has been known to possess profound antibacterial activity against both Gram-positive and Gram-negative bacteria. There are two mechanisms by which nanoceria cause bacterial cell death; namely direct and indirect contact mechanisms (Fig. 2). In direct contact, nanoceria gets directly adsorbed on the bacterial cell and damage the outer cell wall that further leads to the generation of intracellular ROS [117]. Arumugam et al. [58] demonstrated that positively charged nanoceria gets well adsorbed on the bacterial cell due to the electrostatic interaction. Due to this interaction, the cellular proteins become inactive, and nanoceria gets penetrated inside the bacterial cell and deactivating the bacterial enzymes, which generate hydrogen peroxide (ROS). The intracellular ROS so generated further damages DNA, RNA, and protein, which ultimately caused bacterial cell death [59]. Besides, Thill et al. [118] demonstrated that nanoceria might undergo reduction from Ce^4+^ to Ce^3+^ on the cell surface upon adsorption via oxidation of the bacterial wall membrane, thereby leading to oxidative stress.

While in the indirect contact, nanoceria interacts with the environment (intercellular space) around the bacteria and the products (maybe extracellular ROS) formed by this interaction further damage the bacterial cells. This indirect interaction mechanism generally occurs in polysaccharide encapsulated bacteria (the bacteria which have polysaccharide layer around the cell envelope), where nanoparticles have no direct contact with the cell wall. So, in this type of interaction, ROS are generated outside the bacterial cell which enters into the cell through the cell membrane and damages the bacterial cells by degrading nucleic acids and protein which ultimately leads to cell death [117]. Oxygenic photosynthesis is one of the sources of ROS production in cyanobacteria. The ROS which is generated by this process react with Ce^3+^ site at the surface of CNP (which is in close contact with cyanobacteria) and further undergoes oxidative reaction and generates superoxide anions and hydroxyl radicals. These ROS further affect the photosynthetic process and impair membrane integrity by lipid peroxidation, which further leads to cell death [119].

However, in healthy human cells, nanoceria acts as an antioxidant by scavenging ROS. This is due to their ability to show superoxide dismutase (SOD) mimetic activity and catalase (CAT) mimetic activity. For nanoceria, a higher Ce^3+^/Ce^4+^ ratio is necessary for SOD-mimetic activity, whereas for CAT mimetic activity, a higher Ce^4+^/Ce^3+^ ratio is necessary. As nanoceria is capable of interconverting its + 4 and + 3 oxidation states, it is capable of neutralizing both superoxide and hydrogen peroxide on demand [25]. However, in cancer cells, there is acidic pH which promotes SOD mimetic activity of CNPs but inhibits its catalase mimetic activity which results in the accumulation of the huge amount of H_2_O_2_ (ROS) in the cancer cell. So in cancer cells, CNPs showed toxic effects by producing ROS [120]. However, the antibacterial properties of nanoceria depend upon some crucial factors, such as size [121], concentration [58], pH [122], surface coating [123] and surface chemistry [124]. Several researchers studied the antibacterial activity of nanoceria against different strains of bacteria, as mentioned in Table 11.

**Table 11:** Antibacterial activity

S. no	Particle size/morphology	Type of bacteria	Conc.	Observation	References
1.	7 nm/ellipsoidal	*Escherichia coli*	0 to 730 mg/L	A large amount of CNPs was adsorbed on the *E. coli*, showing cytotoxicity on *E. coli*	[118]
2.	140 nm	*Escherichia coli*	10 mg/mL	A drastic decrease in the concentration of *E. coli*	[123]
3.	7 nm 25 nm/truncated octahedral, rhombus or irregular	*Escherichia coli*	10, 100, and 200 mg/L	Direct contact of CNPs with the surface of *E. coli* causes a rise in intracellular ROS level, which shows antibacterial activity	[125]
4.	8–10 nm	*Escherichia coli*	4.3 ppm	Dextran-coated CeO_2_ are non-toxic or exert mild anti-bacterial activity to *E. coli*	[126]
5.	25-50 nm	*Escherichia coli*	5.0 g/L	Under UV irradiation (2 h), CeO_2_ inhibited the growth of *E. coli* cells due to oxidative stress	[127]
6.	100 nm/octahedral or truncated octahedral	*Escherichia coli*	0.075, 0.125, 0.15, 0.175 0.5, 1.0, 1.5, 3.0 and 30 mg/mL	The interaction of nanoceria with non ionic surfactants enhanced their antibacterial activity against *E. coli*	[128]
7.	25–30 nm/elliptically spherical	*Escherichia coli* and *Staphylococcus aureus*	–	Nanoceria inhibited bacterial growth by more than 90%	[59]
8.	10–20 nm	*E. coli*, *K. pneumoniae*, *S. enterica, S. aureus*, and *E. faecalis*	50 mg/mL, 250 mg/mL and 500 mg/mL	Nanoceria disrupted cell membranes of bacteria which led to the irreversible damage to the cell envelope which further results in cell death	[129]
9.	25 nm	*Escherichia coli* (KACC 10005), *S. Typhimurium* (KCCM 40253), *B. subtilis* (KACC 14394) and *E. faecalis* (KACC 13807)	16 µg/mL, 8 µg/mL and 4 µg/mL	Bacterial toxicity is due to the direct interaction between the nanoceria with bacteria which further results in cell death	[124]
10.	5 nm	*Streptococcus mutans*	0.22 mg/mL	Nanoceria seems to be very effective against *S. mutans*	[131]
11.	5 nm/spherical	*Staphylococcus aureus, Streptococcus pneumoniae, Escherichia coli, Pseudomonas aeruginosa, Proteus vulgaris, Klebsiella pneumonia* and *Shigella dysenteriae*	10, 50 and 100 mg	Nanoceria showed strong antibacterial activity	[58]
12.	42 nm/spherical	*Pseudomonas aeruginosa* and *Staphylococcus aureus*	500, 750 and 1000 µg/50 mL	With the increase in the concentration of nanoceria, zone of inhibition also increases in the case of *P. aeruginosa*	[96]
13.	11 nm/spherical	*Staphylococcus* 65 *aureus, Pseudomonas aeruginosa, Escherichia coli,* and *Klebsiella pneumoniae*	1, 3 and 5 mg/disc	Nanoceria exhibited a good antibacterial activity and also showed the inhibition of respective bacterial biofilm formation	[132]
14.	27 nm/spherical	*S. aureus* MTCC-96, *S. pyogenes* MTCC-1926, *P. aeruginosa* MTCC-4673, and *K. pneumoniae* MTCC-109	200 µg	Interaction with nanoparticles causes bacterial cell death due to the generation of reactive oxygen species	[133]
15.	3.5–6.5 nm	*Escherichia coli*	–	Nanoceria significantly inhibited the growth of *E. coli*	[121]
16.	3–4 nm/spherical	*Pseudomonas aeruginosa* and *Staphylococcus epidermidis*	250 μg/mL and 500 µg/mL	Nanoceria possess perfect antibacterial activity against the bacteria at basic pH values as compare to acidic pH values	[122]
17.	40–100 nm/spherical, Cubical and Circular	*Corynebacterium diphtheriae, Sarcina lutea, Escherichia coli, Proteus vulgaris*	20 µl of 25%, 50% and 100% conc.	Nanoceria was very effective against the test organisms and also showed a zone of inhibition for Gram-negative bacteria	[130]

In 2006, Thill et al. [118] studied the antibacterial activity of positive charged ellipsoidal nanoceria with 7 nm size within the concentration range between 1 and 730 mg/L against *E. coli* (Gram-negative bacteria). They observed that a large amount of nanoceria was adsorbed on the outer membrane of *E. coli* by direct contact, which further results in bacterial cytotoxicity. Similar kind of antibacterial activity of nanoceria against *E. coli* has also been reported by other researchers [121, 123, 125–128].

Later, Kannan et al. [59] reported the antibacterial activity of nanoceria (25–30 nm) against *E. coli* (Gram negative) and *Staphylococcus aureus* (Gram positive) bacteria. They observed that nanoceria inhibited the growth of those bacteria by more than 90%. Unnithan et al. [129] assessed the effects of nanoceria doped composite nanofibres on different types of pathogenic bacteria, such as *E. coli* (Gram negative), *K. pneumonia* (Gram negative), *S*. *enterica* (Gram negative), *S. aureus* (Gram positive) and *E. faecalis* (Gram positive). They observed that the number of bacterial colonies was decreased with increasing nanoceria concentrations from 50 to 500 mg/mL. They also found that nanoceria caused disintegration of the bacterial cell membrane, which further resulted in cell death. Later in 2015, Arumugam et al. [58] demonstrated a dose-dependent antibacterial activity of green synthesized nanoceria against different Gram-positive (*Staphylococcus aureus, Streptococcus pneumonia*) and Gram-negative bacteria (*Escherichia coli, Proteus vulgaris, Pseudomonas aeruginosa*, *Klebsiella pneumonia*, and *Shigella dysenteriae*).

After that Reshma and Ashwini [130] studied the antibacterial activity of nanoceria against several Gram-positive (*Corynebacterium diphtheria* and *Sarcinalutea*) and Gram-negative (*Escherichia coli and Proteus vulgaris*) bacteria and showed a significant antibacterial effect of nanoceria. Besides, nanoceria also showed a zone of inhibition for the Gram-negative bacteria.

In the same year, Alpaslan et al. [122] examined the pH, concentration and time-dependent antibacterial activity of dextran-coated nanoceria against *Pseudomonas aeruginosa* (Gram negative bacteria) and *Staphylococcus epidermidis* (Gram-positive bacteria). They observed that nanoceria showed excellent antibacterial activity against both the bacteria at basic pH (pH = 9) compared to acidic pH value (pH = 6) due to positive surface charge and smaller sufficient size. They also found that at pH 9, *S. epidermidis* did not grow at all when treated for 24 h with 500 μg/mL nanoceria, whereas *P. aeruginosa* has grown very slowly at different particle concentrations (250 μg/mL and 500 μg/mL).

Some other researchers [96, 131–133] have also reported the antibacterial activity of nanoceria against different strains of bacteria.

### Antioxidant activity

**Fig. 3:**
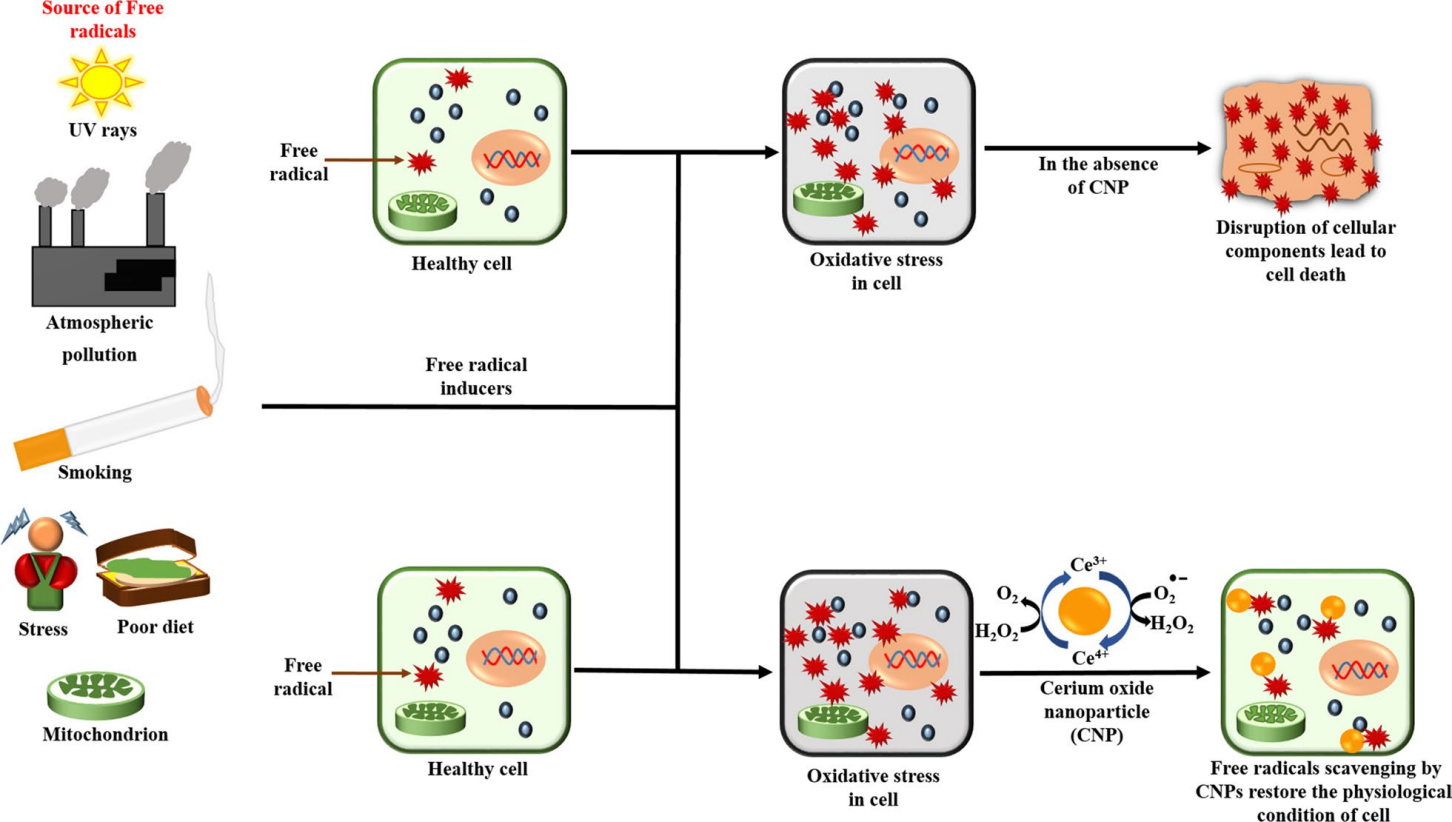
Mechanism of the antioxidant activity of nanoceria. **a** Effect of free radical inducers on healthy cells in the absence of Cerium oxide nanoparticles (CNPs), which ultimately leads to cell death. **b** Effect of free radical inducers on healthy cells in the presence of CNPs, which restores the reasonable condition of the cell

**Fig. 4:**
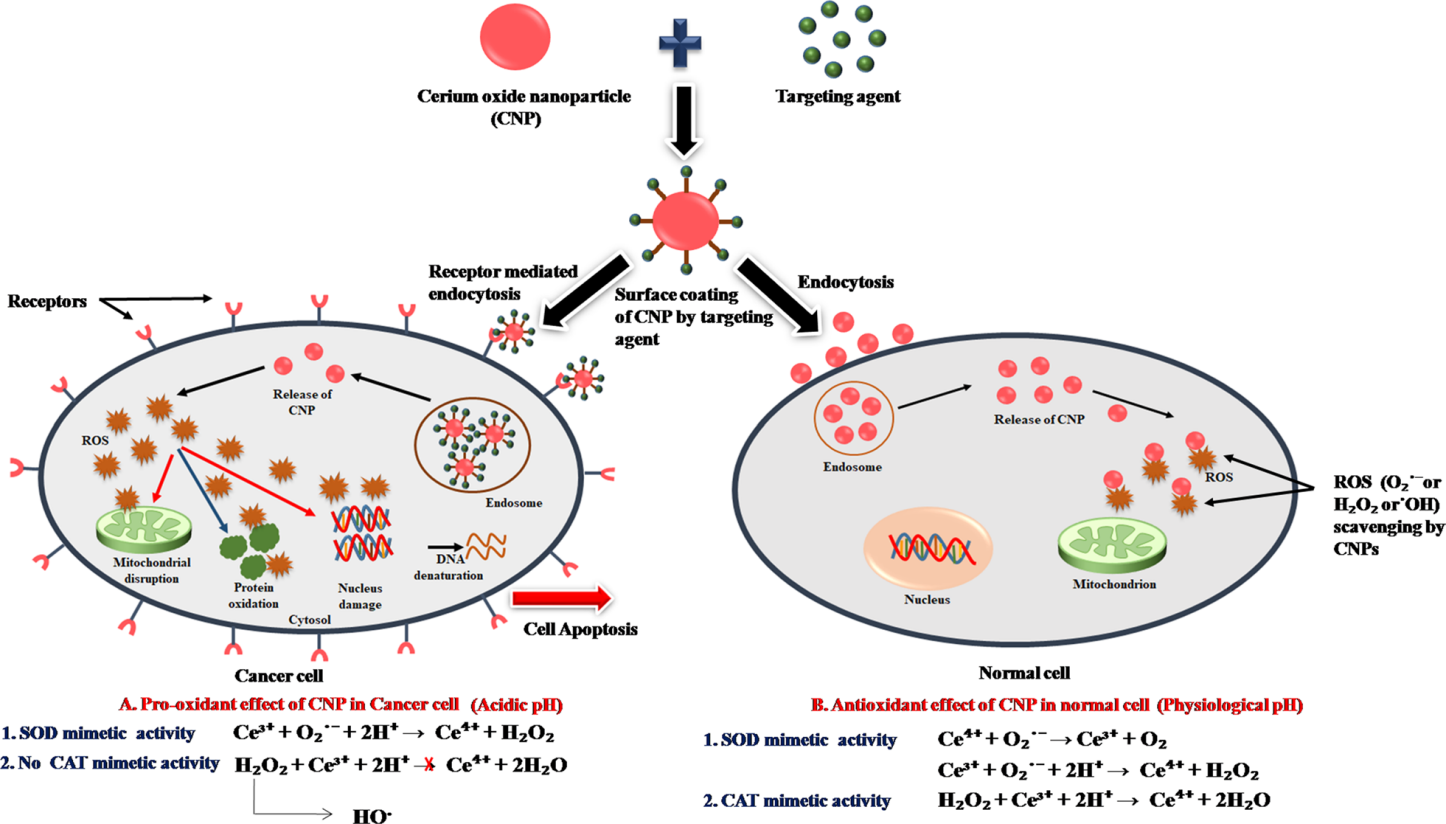
Mechanism of pro-oxidant and antioxidant effect of nanoceria. Cerium oxide nanoparticles (CNPs) showing pro-oxidant effect in the cancer cell by entering into the cell through receptor-mediated endocytosis and get released into the cytoplasm from the endosome. This acidic intracellular pH favors the SOD mimetic activity of CNPs, which reduce superoxide into H_2_O_2_ but inhibits its CAT mimetic activity resulting in the accumulation of the huge amount of H_2_O_2_ in the cancer cell. These ROS further causes mitochondrial disruption, protein oxidation, and denaturation of DNA that results in apoptosis of cancer cell. CNPs showing antioxidant effect in normal cell (having physiological pH) by entering into the cell through endocytosis and scavenging ROS (O_2_·^−^or H_2_O_2_ or ·OH) due to [1] SOD mimetic activity in which superoxide is reduced into H_2_O_2_ and [2] CAT mimetic activity in which H_2_O_2_ gets further degraded into water, hence protecting the normal cell

Nanoceria seems to exhibit vigorous antioxidant activity due to its ability to switch between two oxidation states, Ce^3+^ and Ce^4+^ depending on the environment. This unique redox property of nanoceria can protect different tissues and organs from cellular damage caused by various free radicals or reactive oxygen species (ROS) [76]. The mechanism of the antioxidant activity of nanoceria is shown in Figs. 3 and 4. This antioxidant activity of nanoceria has been examined by different researchers on different cell types, as mentioned in Table 12. In 2007, Das et al. [100] studied the antioxidant activity of nanoceria (3–5 nm) in a serum-free cell culture model of adult rat spinal cord. They observed higher cell survival and less cell death after both 15 and 30 days of culture with medium containing nanoceria as compared to the untreated control cultures. These results showed that nanoceria acts as an antioxidant that scavenges various free radicals present in the culture medium and provides neuroprotection to adult rat spinal cord neurons.

**Table 12:** Antioxidant activity

S. no	Particle size/morphology	Cell type	Observation	References
1.	3–5 nm	Adult rat spinal cord cells	A significant neuroprotective effect on adult rat spinal cord neurons were observed	[100]
2.	4 nm	Cardiomyocytes and human dermal fibroblasts	Dextran-coated nanoceria protects healthy cells against hydrogen peroxide-induced oxidative stress	[35]
3.	3–5 nm	Human colon cells (CRL 1541)	Nanoceria reduce ROS levels and protect healthy human colon cells from radiation-induced damage	[134]
4.	5–8 nm	Cardiac progenitor cells (CPCs)	Nanoceria controls the oxidative stress in CPCs	[139]
5.	20 nm/cubic	Endothelial cells	CeO_2_ reduces intracellular free radicals in endothelial cells and thus helps in controlling cardiovascular diseases	[135]
6.	5–80 nm/cubic	Neuron-like PC12 cells	CeO_2_ scavenged ROS and exerted neuroprotection via regulating genes involved in cellular defense	[140]
7.	< 25 nm/cubic and triangular	A human epithelial lung cell line, BEAS-2B	Pre-treatment of CeO_2_ significantly reduced the intracellular production of ROS induced by KBrO_3_	[136]
8.	5 nm/spherical	NIH3T3 cells	Levan coated CeO_2_ protected NIH3T3 cells against H_2_O_2_-induced oxidative stress	[137]
9.	< 5 nm	Neuron-like SH-SY5Y cells	Showing beneficial effects in terms of neurite development and alignment	[141]
10.	30 nm	Brain tissue samples of rats	CeO_2_ protected against Paraquat-induced neuronal oxidative stress and apoptosis	[138]

Later, Colon et al. [134] studied the radioprotective effect of nanoceria against gastrointestinal epithelium. In their work, they pretreated human colon cells (CRL 1541) with different concentration of nanoceria for 24 h before radiation exposure. They demonstrated that nanoceria acts as a free radical scavenger and protects the human colon cells from radiation-induced oxidative damage via increased cellular production of superoxide dismutase 2.

In 2013, Chen et al. [135] reported the antioxidant activity of nanoceria (20 nm) in endothelial cells against H_2_O_2_. They treated the cultured endothelial cells with different conc. (5, 10, 20, and 40 μg/mL) of nanoceria for 6 to 48 h, and found that the nanoparticles were up taken into the cells through caveolae- and clathrin-mediated endocytosis. After being internalized into endothelial cells, nanoceria effectively counteracts the overproduction of ROS by H_2_O_2_ and decrease the percentage of apoptotic cell death. Based on their observations, they predicted that nanoceria might help in preventing cardiovascular diseases which are caused by oxidative insult. Rubio et al. [136] studied the antioxidant activity of nanoceria in a pulmonary-like cell system against KBrO_3_-induced oxidative stress. They used a human epithelial lung cell line, BEAS-2B, and pretreated those cells with nanoceria before KBrO_3_ exposure. They showed that nanoceria reduced the intracellular ROS production and thus prevented cell death. Kim et al. [137] analyzed the antioxidant activity of levan coated nanoceria in NIH3T3 cells against H_2_O_2_. Levan and their derivatives are well known for their antioxidant, anti-tumor and anti-inflammation properties, and levan coating also confer stability and water solubility to nanoceria. They demonstrated that conjugation of levan with nanoceria exert a synergistic antioxidant effect against H_2_O_2_ stimulated NIH3T3 cells.

Recently, Ranjbar et al. [138] used brain tissue samples of rats to examine the antioxidant activity of nanoceria against paraquat (PQ) induced brain injury. They observed that nanoceria enhanced the antioxidant defense mechanism via increasing the total thiol content and total antioxidant capacity, thereby decreased the lipid peroxidation, oxidative DNA damage as well as caspase 3 activity. Besides, the mRNA levels of Nestin and Neurod1 has also been increased in the brain samples upon nanoceria treatment. Therefore, nanoceria possessed antioxidant and neuroprotective effect against paraquat-induced neuronal oxidative stress and apoptosis in vivo. The antioxidant activity of nanoceria has also been reported by several other researchers [35, 139–141].

### Anticancer activity

In addition to the antibacterial and antioxidant activity, nanoceria is also known to exhibit profound anticancer potential. It provides cytoprotection towards healthy cells from ROS but kills cancer cells via inducing ROS formation. The mechanism of the anticancer activity of nanoceria is depicted in Fig. 4. Cancer cells have a higher acidic environment compared to healthy cells due to an increased rate of glycolysis and lactic acid production [35]. In the acidic environment (low pH), the antioxidant (cytoprotective) activity of nanoceria is lost, and it acts as a prooxidant which causes oxidative stress by producing ROS and thus induces cell death/apoptosis [25]. This activity of nanoceria has been studied by many researchers against different cancer cell lines, as illustrated in Table 13.

**Table 13:** Anticancer activity

S. no	Size (nm)	Cell line	Observation	References
1.	20	Human lung cancer cells (A549 cells)	Free radicals were generated on the exposure of 3.5 to 23.3 µg/mL nanoceria which causes oxidative stress and cytotoxic effect in the cancer cells	[142]
2.	100–200	Human prostate cancer cell line (PC-3)	Nanoceria showed cytotoxicity in prostate cancer cells but was non-toxic in normal cells at the conc. of 5 mg/mL	[48]
3.	3–5	Ovarian cancer cells (A2780) and A2780 xenograft murine model	Conc. between 25 and 50 µM showed an anti-angiogenic effect in ovarian cancer cells and reduced tumor size in vivo	[75]
4.	3–5	Human colon cancer cells (HCT 15)	Conc. between 10 and 100 µM resulted in a significant reduction of cell viability via increasing ROS levels	[144]
5.	< 25	Human neuroblastoma cell line (IMR32)	Nanoceria exposure generated ROS that induced oxidative stress, which leads to cytotoxicity and genotoxicity in IMR32 cells at higher conc. (> 100 µg/mL)	[145]
6.	10	Ovarian cancer cells (A2780) and A2780 xenograft murine model	Folic acid tagged nanoceria showed significant inhibition in viable cells in A2780 cells within the conc. range 10–100 μM, and reduced tumor size in vivo	[143]
7.	30	Fibrosarcoma cell line (WEHI164)	Conc. of nanoceria ≥ 15.63 µg/mL showed toxicity effects in cancer cells via increasing ROS levels and apoptosis	[146]

In 2006, Lin et al. [142] studied the anticancer effect of nanoceria (20 nm) in human lung cancer cells (A549 cells), which were exposed to different conc. (3.5, 10.5, and 23.3 µg/mL) of nanoceria for 24, 48, and 72 h. They showed a dose and time-dependent cytotoxicity of nanoceria towards A59 cells via oxidative stress. Renu et al. [48] demonstrated that nanoceria with conc. Of 5 mg/mL showed cytotoxicity towards prostate cancer cell line (PC3), but nontoxic towards normal mouse fibroblast cell line (L929). They prepared nanoceria by two different methods; hydrolysis (HL) method resulted in the formation of nanoceria with + 3 oxidation state and hydrothermal (HT) method resulted in the formation of nanoceria with + 4 oxidation state. They also observed that HT-nanoceria were more cytotoxic towards PC3 cells compared to HL-nanoceria because of their increased cellular uptake. In 2013, Giri et al. [75] studied the anticancer effects of nanoceria in ovarian cancer cells (A2780) and A2780 xenograft tumor mice model. They treated the A2780 xenograft mice with 0.1 mg/kg body weight of nanoceria intra-peritoneally every third day till up to 30 days and compared the tumor growth with the untreated mice. They observed that the tumor weight and the abdominal circumference in the mice treated with nanoceria were significantly reduced as compared to the untreated mice. They showed that nanoceria had a tendency to inhibit metastasis and angiogenesis in ovarian cancer cells and thus attenuated ovarian tumor growth. Hijaz et al. [143] also demonstrated that folic acid tagged nanoceria possessed significant anticancer activity against ovarian cancer cells (A2780) and A2780-xenograft tumor mice. In their in vivo experiment, they treated A2780 xenografts tumor mice with 0.1 mg/kg body weight of either bare nanoceria or folic acid tagged nanoceria. They observed that folic acid tagged nanoceria was more effective in restricting the tumor growth in mice via targeted delivery into tumor cells.

Cytotoxic effects of nanoceria in human colon cancer cells (HCT 15) were observed in a dose- and time-dependent manner by Jana et al. [144]. They showed that the exposure of nanoceria with conc. between 10and 100 µM (for 24, 48 and 72 h) resulted in cytotoxicity via the production of ROS which subsequently depolarised the mitochondrial membrane and initiated apoptosis. Kumari et al. [145] checked the cytotoxic and genotoxic effects of both cerium oxide nanoparticles (CNPs) and cerium oxide microparticles (CMPs) in the human neuroblastoma cell line (IMR32) upon treatment with different conc. (100 and 200 µg/mL) for 24 h. They observed that the cells incubated with CNPs showed much higher production of intracellular ROS and induced higher cytotoxicity because of increased cellular uptake as compared to CMPs. Recently, in 2018, Nourmohammadi et al. [146] studied the anticancer activity of nanoceria against fibrosarcoma cell line (WEHI164), which showed dose-dependent cytotoxicity of nanoceria. They observed that nanoceria exposure with conc. ≥ 15.63 µg/mL showed profound cytotoxicity in cancer cells (WEHI164), whereas very little toxicity was observed in normal (L929) cells even at conc. above 250 µg/mL. They further demonstrated that nanoceria exhibit toxicity in cancer cells by generating ROS which induces apoptotic cell death.

### Drug/gene delivery

**Fig. 5:**
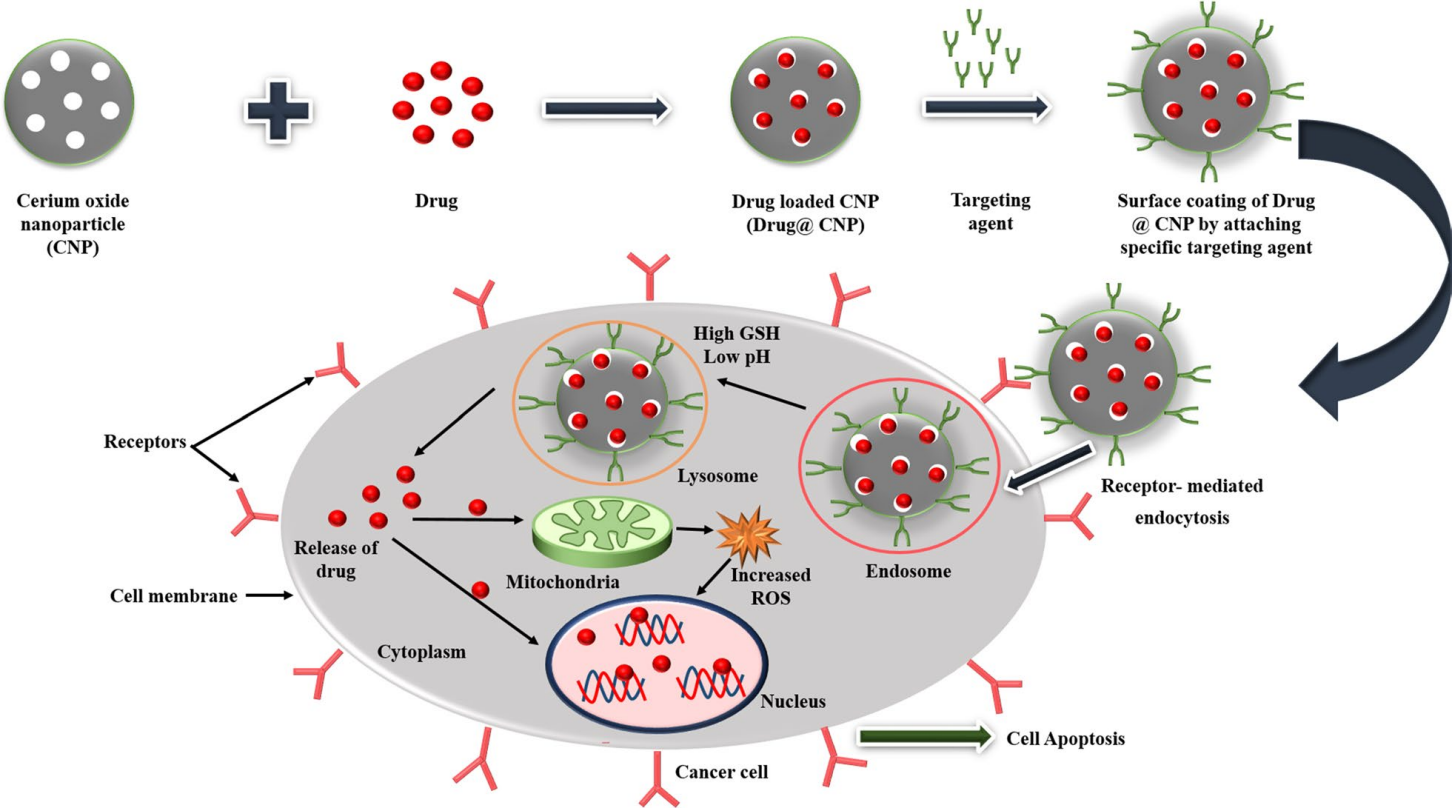
Schematic diagram of drug delivery activity of nanoceria in the cancer cell. Drug @ CNP coated with specific targeting agent is up taken by the cell through endocytosis. Due to low pH and high GSH in endosome, and lysosome drug is released into the cytoplasm and then enter either into nucleus directly and bind with DNA that causes its denaturation or in mitochondria which increased the production of ROS that further attack the nucleus and causes denaturation of DNA, which ultimately lead to cell death

Recently, nanoceria has widely been used in the biomedical field as an effective and targeted drug and gene delivery vehicle. This drug-delivery application of nanoceria in cancer cells shows a synergistic anticancer effect due to its inherent cytotoxicity towards cancer cells (Fig. 5). The use of nanoceria as a drug and gene delivery systems has been studied by some researchers, as mentioned in Tables 14 and 15.

**Table 14:** Drug delivery

S. no	Name of drug	Cell type	Observation	References
1.	Carboxybenzene sulphonamide	None	Showed an inhibition of hCAII which is very useful in the treatment of glaucoma	[115]
2.	Camptothecin	Human pancreatic cancer cell lines BxPC-3 cells	Increase in the conc. of camptothecin-loaded nanoceria decreases the cell viability of BxPC-3 cells	[147]
3.	Chlorin e6	Human breast cancer cells (MCF-7/ADR) and MCF-7/ADR xenograft murine model	Showed photodynamic therapy in drug-resistant breast cancer cells and tumor in vivo	[148]
4.	Doxorubicin	Human Ovarian cancer cell lines A2780, SKOV-3, and CAOV-3	Higher cell proliferation inhibition and apoptosis compared with free DOX	[116]
5.	Doxorubicin and Hsp90 inhibitor ganetespib (GT)	A549 cells	Co-delivery of Dox and GT using Nanoceria showed more than 80% of NSCLC death within 48 h of incubation	[33]
6.	Curcumin	Neuroblastoma cell lines: IMR-32, SMS-KAN, SK-N-AS, and LA–N-6	Induce substantial cell death in neuroblastoma cells	[34]
7.	Doxorubicin	Human liver cancer cells (HepG2 cells)	Showed a synergistic anticancer effect on cancer cells	[52]

**Table 15:** Gene delivery

S. no	Name of gene	Cell type	Observation	References
1.	Luc gene, EGFP gene, and RFP gene	HEK293, MCF-7 and Hep G2 cells	CeO_2_/DODAB nanovectors could transfect genes in vitro and in vivo without causing any toxic effect	[149]

In 2007, Patil et al. [115] studied the utility of nanoceria as a potential drug delivery device by attaching carboxybenzene sulfonamide (an inhibitor of the human carbonic anhydrase enzyme) along with a fluorophore (carboxyfluorescein) to nanoceria using epichlorohydrin as a linker molecule. They demonstrated that such conjugated nanoceria could be used for the inhibition of human carbonic anhydrase enzyme (hCAII), which is very useful in the treatment of glaucoma. Later, Muhammad et al. [147] developed a redox-responsive mesoporous silica nanoparticle (MSN) based camptothecin (an anticancer drug) delivery vehicle for active transport of drug into human pancreatic cancer cells (BxPC-3 cells). They further capped the pores of the drug-loaded MSN using nanoceria to inhibit the premature leakage of the drug. However, the nanoceria lids were dissolved upon exposure to antioxidant molecules, like vitamin-c and GSH, and triggered the redox-responsive drug release. They also observed that camptothecin loaded and nanoceria capped MSN exhibit dose and time-dependent cytotoxicity in BxPC-3 cells due to its active intracellular uptake, dissolution of nanoceria lid in highly reducing cellular environment and release of the encapsulated drug. Li et al. [148] reported a nanoceria mediated delivery system by conjugating a photosensitizer, chlorin e6 (Ce6) and folic acid (FA) on polyethylenimine-PEGylation ceria nanoparticles (PPCNPs) for targeted photodynamic therapy to treat drug-resistant human breast cancer cells, MCF-7/ADR and xenograft murine model. They observed that upon near-infrared (NIR) irradiation, PPCNPs-Ce6-FA generate ROS, which leads to a reduction in *P*-glycoprotein expression, lysosomal membrane permeabilization, and cytotoxicity towards drug-resistant breast cancer cells even at shallow doses. They further treated the mice with free Ce6, PPCNPs-Ce6 or PPCNPs-Ce6/FA intravenously and irradiated with NIR at the tumor site. The mice treated with PPCNPs-Ce6/FA showed significant tumor growth inhibition in drug-resistant MCF-7/ADR tumors with a decrease in the ~ 96% volume of the tumor via the production of ROS. On the other hand, the mice treated with PPCNPs-Ce6 or free Ce6 after irradiation showed weaker or no effect on tumor growth. However, without irradiation, the administration of PPCNPs-Ce6/FA showed no tumor regression in the mice.

In 2017, Das et al. [116] reported nanoceria-mediated drug delivery of doxorubicin in human ovarian cancer cells. They found that doxorubicin-loaded nanoceria (CeO_2_/DOX) possessed excellent drug-loading content (22.41%) and drug release behavior at the acidic and high reducing environment as well as increased cellular uptake and retention of the drug as compared to free doxorubicin. They further showed that doxorubicin-loaded nanoceria exhibited a higher degree of apoptosis and cell proliferation inhibition than free doxorubicin in human ovarian cancer cells.

In 2017, Sulthana et al. [33] reported about polyacrylic acid (PAA) coated functional nanoceria loaded with a combination of two therapeutic drugs; Hsp 90 inhibitor, ganetespib (GT) and doxorubicin (doxo) for the treatment of non-small-cell lung cancer (NSCLC). They further conjugated the nanoceria with folic acid to target folate receptors expressing NSCLC. They observed that this combination therapy results in the death of more than 80% of NSCLC cells within 48 h of incubation, whereas around 40% of cell death occurred in the case of single drug-loaded nanoceria. Later, Kalashnikova et al. [34] used dextran-coated nanoceria loaded with curcumin for the treatment of human childhood neuroblastoma and exploring their anti-cancer activities in neuroblastoma models, including both MYCN-amplified and non amplified cell lines. They demonstrated that dextran-coated nanoceria loaded with curcumin-induced significant cytotoxicity in neuroblastoma cells while sparing healthy cells. More recently, Zhang et al. [52] developed a multifunctional and pH/GSH dual-responsive drug delivery system (MDDS) using porous cerium oxide nanorods loaded with doxorubicin for drug delivery in human liver cancer cells (HepG2 cells). This MDDS was further conjugated with lactose derivatives which recognize the asialoglycoprotein receptors present on the surface of liver cancer cells. They observed that the as-prepared drug-loaded and targeted MDDS showed a synergistic anticancer effect in HepG2 cells due to low intracellular pH and high GSH level inside the lysosomes in cancer cells.

**Fig. 6:**
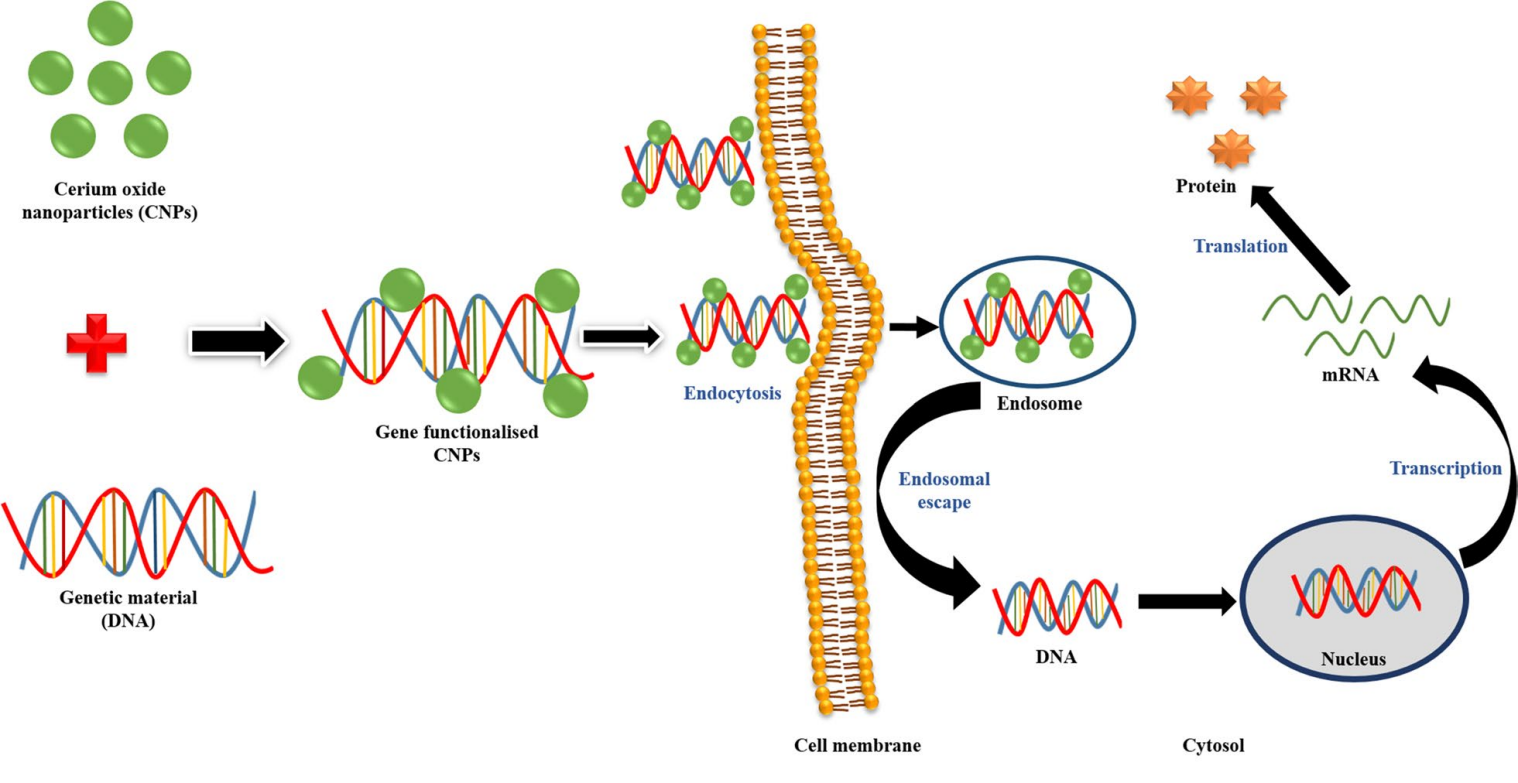
Schematic representation of gene delivery activity of CNPs. Intracellular uptake of gene functionalized CNPs through endocytosis form endosome from which gene (DNA) is released into the cytosol, and then enter into the nucleus which forms mRNA by transcription and mRNA further form protein by translation

The first report of gene delivery using nanoceria as a carrier was published by Das et al. [149] in 2016. They developed dimethyldioctadecylammonium bromide (DODAB)—nanoceria hybrids as an efficient non-viral gene delivery vector for the transfection of plasmid DNA (pEGFPN1) in several cells lines, HEK293, MCF-7, and HepG2. They found that the overall vector performance of DODAB-modified CeO_2_ nanohybrids (CeO_2_/DODAB) was comparable with lipofectamine 2000 and DOTAP(1,2-dioleoyloxy-3-trimethylammonium propane) and higher than calcium phosphate and DEAE-dextran used for transfecting small (4.7 KD) plasmids. The higher gene delivery efficiency of this nanohybrid vector was further supported by the improved cellular uptake of the nanovector/DNA complexes through clathrin- and caveolae-mediated endocytosis and their subsequent release from the endosomes. Further, CeO_2_/DODAB nanovectors were also checked for their in vivo transfection efficiency via injecting the plasmid/nanovector complexes into the posterior tibialis muscles of mice. They observed a 3.5 times higher fluorescence intensity with CeO_2_/DODAB compared with the naked DNA treated groups after 72 h, and the transfection efficiency was around 17% less than the commercial in vivo-jeiPEI reagents. The results obtained from their study showed that nanoceria possessed great potential for its use as a carrier in gene delivery as represented in Fig. 6.

### Beneficial effect of nanoceria against diabetes and its associated complications

**Fig. 7:**
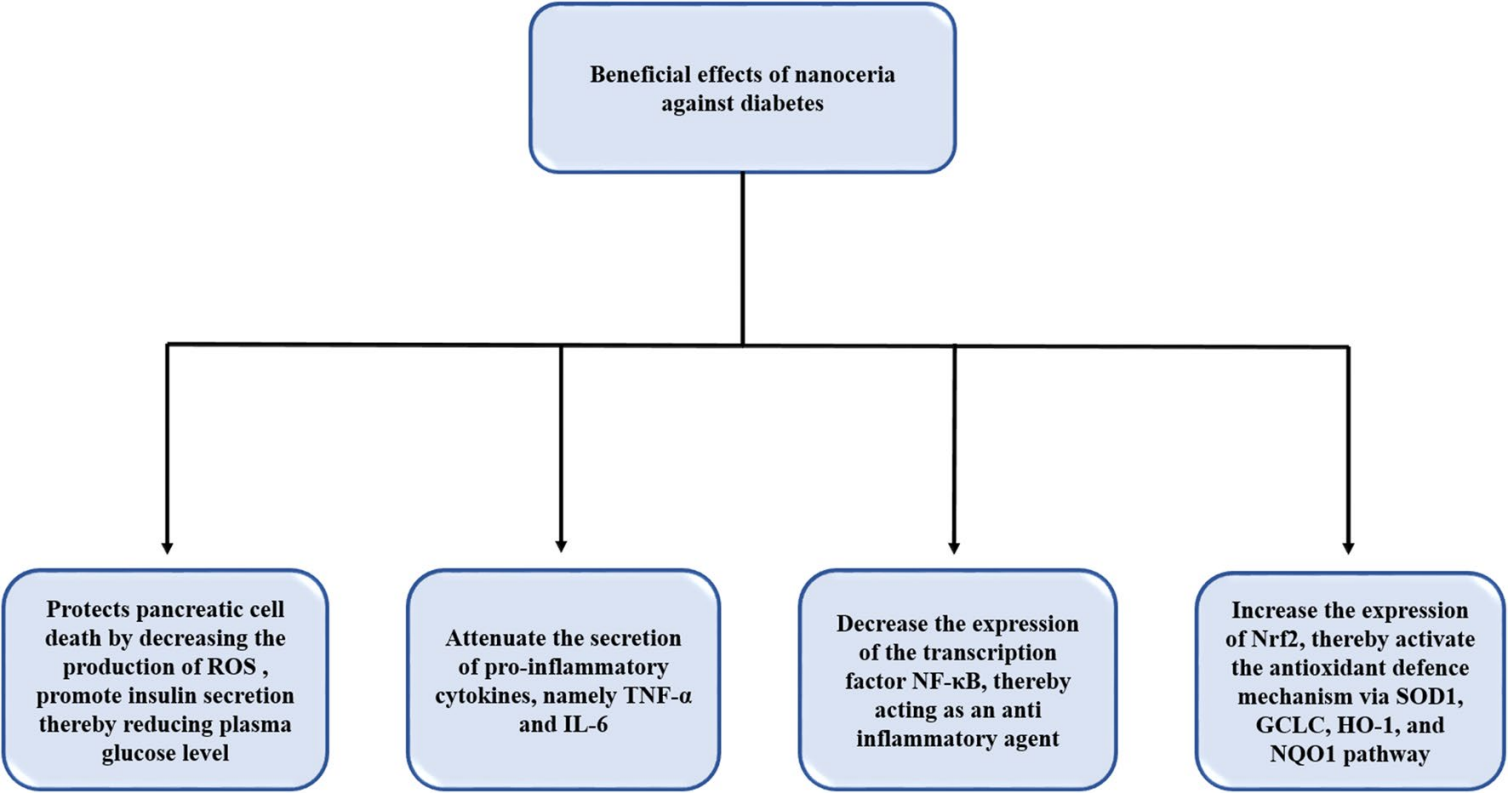
Anti-diabetic activity of nanoceria

Diabetes is one of the most serious and leading health disaster of the 21st century. It is characterized by an increase in plasma glucose concentration due to complications in either insulin secretion, insulin action, or both. The prevalence of diabetes is increasing at an alarming rate causing severe health and economic burden to the patients and the society at large. According to the 2017 reports of International Diabetes Federation (IDF), approximately 425 million people around the world were found to be diabetic and is predicted to increase to 629 million by 2045 (IDF Diabetes Atlas 8th Edition) [150]. Over time, diabetes can lead to several organs and tissue damage ranging from cardiac dysfunction, blindness, renal dysfunction, nerve damage, erectile dysfunction, etc. Oxidative stress has been recognized as a significant contributing factor in the pathogenesis of diabetes and its associated complications [151]. Everyday factors, like unhealthy diets, obesity, aging, etc. have been found to contribute to an oxidative environment, which may cause a decrease in insulin sensitivity, impaired glucose tolerance, and an increase in insulin resistance. Due to its potential antioxidant nature, CNP is gaining acceptance as a prospective therapeutic material against hyperglycemia and its associated complications in diabetes (Fig. 7). The anti-diabetic effect of CNP has been studied by several researchers, as depicted in Table 16.

**Table 16:** Anti-diabetic Effect

S. no.	Particle size	Model	Observation	References
1.	180 ± 15.54 nm by DLS and 90 ± 9.5 nm by SEM	STZ-treated diabetic mice were treated with CNPs (0.2 and 2 mg/kg bw, i.p., 28 days)	CNPs treatment decreased the glucose levels, lipid peroxidation, secretion of pro-inflammatory cytokines, and NF-κB protein expression and increased the insulin levels and glutathione concentrations	[152]
2.	–	HepG2 cells were treated with 50 mM CNPs against high glucose (50 mM) exposure	Treatment with CNPs significantly decreased the high glucose-induced cytotoxicity, ROS formation, lipid peroxidation, and increased intracellular glutathione	[153]
3.	–	STZ-treated diabetic animals were administered CNPs (30 mg/kg bw, daily, i.p., 2 weeks)	CNPs administration alleviated the plasma glucose levels and the deleterious effects of diabetes on the sperm potential fertility, sperm parameters, DNA integrity, and Nrf2 expression levels	[154]
4.	–	STZ-treated diabetic animals were administered CNPs (30 mg/kg bw, daily, i.p., 2 weeks)	CNPs administration increased the total antioxidant capacity via upregulating Nrf2 mediated increase in the mRNA expressions of antioxidant genes, namely GCLC, HQ-1, and NQO1	[155]
5.	–	STZ-treated diabetic mice were treated with CNPs (60 mg/kg bw, 16 days)	CNPs treatment significantly prevented embryonic oxidative stress and pathologic changes in diabetic mice	[157]
6.	–	Isolated pancreatic islets were pre-treated with CNPs (10, 100, 1000 nM)	Treatment with CNPs increased the cell viability, secretion of insulin, and ATP/ADP ratio and reduced the ROS level	[158]
7.	–	Isolated pancreatic islets were pre-treated with CNPs (200 µM) against H_2_O_2_ (50 µM, 2 h)	Pre-treatment with CNPs attenuated the ROS formation, caspase-3 activity, and apoptotic cell death and increased cell viability, glucose-induced ATP production, and glucose-stimulated insulin secretion	[159]

Khurana et al. [152] examined the antidiabetic effect of cerium oxide nanoparticles (CNPs) by using multiple low doses of streptozotocin (STZ)-induced type 1 diabetic mice. Results demonstrated that treatment with CNPs (0.2 and 2 mg/kg body weight, i.p., 28 days) significantly reduced the plasma glucose levels and improved the glucose tolerance in a dose-dependent manner in diabetic mice. Moreover, CNPs administration also caused an increase in GSH levels and a decrease in lipid peroxidation and NO levels compared to those seen in the pancreatic tissues of the diabetic mice, which suggests a beneficial role of CNPs in preventing the high glucose-induced oxidative and nitrosative stress in diabetic mice. The intervention of CNPs also led to attenuation of the secretion of pro-inflammatory cytokines, namely TNF-α and IL-6 and an increase in plasma insulin levels in the diabetic mice. Further mechanistic studies demonstrated a decrease in NF-κB and an increase in Nrf2 and SOD1 protein expression in CNPs treated pancreatic tissues of diabetic mice suggesting a role of NF-κB/Nrf2 signaling pathway in mediating the anti-inflammatory and anti-oxidant potential of CNPs. Both TUNEL assay and immunohistological analysis of cleaved caspase three also demonstrated the reduction of DNA fragmentation and apoptotic cell death in CNPs treated pancreatic tissues of the diabetic mice. Taken all together, the authors reported a potential antidiabetic role of CNPs in STZ-induced type 1 diabetic mice.

The beneficial effect of CNPs against hyperglycemia-induced oxidative damage has been further investigated by using high glucose-treated HepG2 cells [153]. Results showed that high glucose treatment caused a decrease in cell viability, an increase in ROS production and lipid peroxidation, and a decrease in intracellular GSH levels. Pre-treatment with CNPs (0–200 mM) significantly protected the cells from high glucose-induced cytotoxicity, and a maximum protective effect was observed at a dose of 50 mM against 24 h exposure of high glucose. Treatment with CNPs also reduced the intracellular ROS production and lipid peroxidation and increased the GSH levels in high glucose-treated HepG2 cells suggesting a beneficial role of CNPs against hyperglycemia-induced oxidative impairment in HepG2 cells.

Oxidative stress plays an essential role in the pathophysiology of diabetes associated with impaired reproductive function. Artimani et al. [154] examined the impact of CNPs on the sperm parameters, spermatogenesis, and Nrf2 protein expression in the testicular tissues of the STZ-treated diabetic rodents. It has been observed that treatment with CNPs (30 mg/kg bw, daily, i.p., 2 weeks) significantly decreased the plasma glucose levels and increased the sperm count and mortality compared to those seen in STZ-treated diabetic animals. Furthermore, CNPs administration significantly elevated the plasma levels of LH and FSH but not testosterone in diabetic animals. Histological findings revealed that CNPs treatment significantly declined seminiferous tubule abnormality, increased the number of Leydig cells, and improved the testicular physiology. An increase in Nrf2 mRNA expression and a decrease in sperm head DNA fragmentation in diabetic animals after CNPs treatment also supported the beneficial role of CNPs in preventing the reproductive dysfunction associated with diabetes. In another study, Artimani et al. [155] further examined the impact of CNPs administration on the oxidative stress status and expression of anti-oxidative genes related to Nrf2 signaling pathway, namely HO-1, GCLC, and NQO1 in testes tissues of STZ-induced diabetic rats. Results demonstrated that CNPs administration (30 mg/kg body weight, daily, i.p., 2 weeks) alleviated the deleterious effect of diabetes via increasing the total antioxidant capacity and decreasing the total oxidative stress in the testicular tissues of the diabetic rats. Treatment with CNPs also caused a decrease in the mRNA expression of the antioxidant genes, HO-1, GCLC, and NQO1 compared to those seen in the diabetic group and the authors also a positive correlation between Nrf2 and HO-1, GCLC, or NQO1, which suggests a critical role of Nrf2-dependent antioxidant response in mediating the underlying mechanisms associated the protective effect of CNPs in diabetes testes.

Gestational diabetes increases the incidences of congenital anomalies, premature birth, fetal macrosomia, neonatal hyperglycaemia, and infant death [156]. Oxidative stress has been implicated in the pathogenesis of diabetic embryopathy. Recently, Vafaei-Pour et al. [157] examined the embryo-protective effect of CNPs by using STZ-induced diabetic female mice model. Results showed that administration of CNPs (60 mg/kg body weight, 16 days) significantly inhibited the embryonic oxidative stress in diabetic mice as revealed by the decrease in ROS formation, lipid peroxidation, and protein carbonylation and the increase in GSH level and catalase activity compared to those seen in the diabetic group. Moreover, CNPs treatment partially restored the hyperglycemia-induced malformation in visceral and spinal of the embryo. Combining all, the results suggest a beneficial role of CNPs treatment in preventing diabetic embryopathy.

Pancreatic islet transplantation has been recognized as a promising treatment strategy for patients with insulin dependent diabetes mellitus (IDDM). Despite recent improvements, the loss of healthy islet at the end of the operation is the main restriction, and this may be due to the generation of excessive oxidative stress during isolation and transplantation procedures. Inherently low levels of antioxidant capacity, scarcity of vascular system, and hypoxic ischemia are playing an essential role in making these cells vulnerable for oxidative damage. Abdollahi et al. [158] have examined the beneficial effect of CNPs combined with sodium selenite in improving the biochemical function of isolated rat pancreatic islets. It has been observed that treatment with CNPs (100 nM) along with sodium selenite nanoparticles significantly increased the cell viability, insulin secretion, and ATP/ADP ratio and decreased the ROS production in the isolated rodent pancreatic islets compared to those seen in CNPs or sodium selenite alone treated cells. These results suggest a beneficial role of CNPs along with sodium selenite in improving the transplantation outcome of pancreatic islets. In another study, Abdollahi et al. [159] further investigated the beneficial role of CNPs along with yttrium oxide nanoparticles in preventing the H_2_O_2_ induced oxidative impairment in isolated rodent pancreatic islets. Results demonstrated that CNPs treatment (200 µM) with or without yttrium oxide against H_2_O_2_ exposure (50 µM, 2 h) significantly reduced the intracellular ROS formation and increased the cell viability, glucose-induced ATP production, and glucose-stimulated insulin secretion in isolated pancreatic islets. Interestingly, treatment with CNPs with or without yttrium oxide also caused a reduction in caspase-3 and caspase-9 activities followed by a decrease in apoptotic cell death (examined by Hoechst staining) in H_2_O_2_-treated cells. In conclusion, results suggested that treatment with CNPs with or without yttrium oxide may protect the pancreatic cell death via improving the oxidative stress-mediated apoptotic pathway.

### Nanoceria directed stem cell differentiation for tissue regeneration

Nanoceria has also been used as an excellent therapeutic agent in tissue repair and regeneration. The tissue repairment ability of nanoceria is due to its ROS scavenging property and angiogenic potentials. Further, nanoceria can also induce stem cell differentiation, which helps in tissue regeneration [160]. Karakoti et al. [77] studied the role of nanoceria in bone regeneration by using human mesenchymal stem cells (HMSCs). They incorporated nanoceria in bioactive glass foam scaffolds which were further compared with bioactive scaffolds without nanoceria. They showed that the as-prepared nanoceria was non-toxic to the HMSCs, and the bioactive scaffolds containing nanoceria increased the production of collagen and enhanced the osteoblastic differentiation of HMSCs after 10 days of culture because of its oxygen buffering property. In 2015, Zhang et al. [161] used primary mouse bone marrow stromal cells (BMSCs) and examined the effect of nanoceria on the osteogenic and adipogenic differentiation. They showed that nanoceria is non-toxic towards BMSCs when incubated for 24 and 72 h. They further showed that nanoceria exhibit both concentration and time-dependent differentiation of the BMSCs into osteoblasts and adipocytes. Recently, Marino et al. [141] in 2017 prepared highly-aligned nanocomposite fibers of gelatin/nanoceria as antioxidant scaffolds for nerve regeneration. They examined the effect of these nanofibres on neuron-like SH-SY5Y cells. They showed that the as-prepared substrates were able to sustain the growth and differentiation of neuronal cells due to the scaffolds topography as well as antioxidant nature.

All these studies indicated that nanoceria could be used as a composite scaffold for the regeneration of bone, adipose, and nerve tissues, as mentioned in Table 17.

**Table 17:** Tissue regeneration activity

S. no	Therapeutic target tissue	Cell type	Observation	References
1.	Bone	Human mesenchymal stem cells (HMSCs)	Bioactive scaffolds containing nanoceria increased the production of collagen and enhanced the osteoblastic differentiation of HMSCs	[77]
2.	Bone and soft tissue	Bone marrow stromal cells (BMSCs)	Nanoceria increased the viability of BMSCs and also showed concentration and time-dependent proliferation, osteogenic, and adipogenic differentiation of BMSCs	[161]
3.	Nerve	Neuron-like SH-SY5Y cells	Gelatin/nanoceria nanocomposite fibers improved the growth and differentiation of neuronal cells	[141]

## Adverse effects of nanoceria

Despite showing various promising biomedical applications, nanoceria also shows some toxic effects, which are currently a significant concern. Therefore, risk assessment of workplace exposure to these engineered nanoparticles should be a significant issue. Besides, risk assessment can also be applied to the product in which these nanoparticles are incorporated.

The toxicity of nanoceria depends upon various factors, such as particle size, preparation method, cell type, dose/concentration, exposure time, and exposure route [162]. Aalapati et al. [163] studied the toxic effects and bio-accumulation of nanoceria in CD1 mice and observed that nasal inhalation exposure of nanoceria results in pulmonary and extrapulmonary toxicity in CD1 mice. Wu et al. [164] studied the size-dependent toxicity of nanoceria on mice after repeated intranasal instillation. Two different sized nanoceria (7 nm and 25 nm) were used in their experiment to study the toxic effect of nanoceria in the lung, liver, spleen, kidney, and brain of mice. They observed size-dependent pulmonary damage for nanoceria. On the other hand, both sized nanoceria showed similar systemic toxicity to other organs.

In 2014, Kumari et al. [165] analyzed the genotoxicity by providing repeated oral administration of 30, 300 and 600 mg/kg body weight per day of nanoceria and cerium oxide microparticles for 28 days in Wistar rats. They observed that nanoceria exhibited toxic effects without any severe distress symptoms and mortality at medium and high doses. On the other hand, microparticles did not show any toxicity. They observed that long-term exposure of nanoceria at higher conc. caused genetic damage (DNA damage in peripheral blood leukocytes and liver), histological changes (alterations in liver, spleen, and brain) and biochemical alterations (alterations in lactate dehydrogenase and alkaline phosphatase activity in serum and also reduction in glutathione content in liver, kidney, and brain) in rats.

Nanoceria also shows immunotoxicological effects by interfering with the immune system. Ranjbar et al. [166] studied the dose-dependent effect of nanoceria on rat liver by giving them the intraperitoneal injection. In their study, they observed that nanoceria with a dose of 15 mg/kg body weight acted as an antioxidant, thereby preventing oxidative stress. Besides, it also decreased the level of inflammatory cytokines, such as IL-17 and TNF-α in the rat liver, which suppressed the inflammatory response. In contrast, they also observed that nanoceria with doses of 30 and 60 mg/kg body weight showed an increase in the level of IL-17 and TNF-α, thereby developed inflammation and immunotoxicity in the rat liver tissue.

In 2018, Gagnon et al. [167] observed that the exposure of nanoceria in natural water caused immunotoxicity in rainbow trout fish. They found that when the fishes were exposed to the water, nanoceria got accumulated in their gills. The accumulation was highest in green water that possesses higher pH, higher conductivity, and contained less total organic carbon as compared to brown water. Therefore, nanoceria induced phagocytosis in fish exposed to tap and green water but not in brown water.

## Conclusion and future perspectives

Nanoceria has been found to possess great potential for a broad range of applications, particularly in the biomedical field. In this review, different methods used for the synthesis of nanoceria have been discussed along with its biomedical applications. Among the existing methods, nowadays, green synthesis method has been widely focused by the researchers to synthesize nanoceria that uses biocompatible stabilizers and produce less toxic effects. These methods are considered very important in order to understand the actual physiochemical properties of nanoceria. The paper exclusively focuses on the biomedical applications of nanoceria, in which its antibacterial, antioxidant, anticancer, drug/gene delivery, antidiabetic, and tissue regeneration activities have been discussed. Nanoceria has been found to exert a profound antibacterial effect against different strains of bacteria. The usefulness of nanoceria depends upon its inherent property of showing variable oxidation states, due to which it can act as an excellent antioxidant agent and protect the healthy cells from oxidative stress.

On the other hand, in cancer cells (under low pH environment), it acts as a pro-oxidant by producing reactive oxygen species and kills cancer cells. Nanoceria has also been used as a carrier for drug and gene delivery into specific target cells and can be helpful in the treatment of various types of diseases. Nanoceria confers protection to the ROS-mediated damage of pancreatic beta cells, thereby could promote insulin secretion, reduced plasma glucose levels and thereby showed a beneficial effect against diabetes-induced organ pathophysiology via decreasing the level of intracellular ROS. Besides, nanoceria also exhibits excellent potential in the field of tissue regeneration by directed stem cell differentiation.

Although there are numerous reports on antibacterial and antioxidant activities of nanoceria, there is minimal research on its anticancer, drug/gene delivery, anti-diabetic, and tissue regeneration applications. So, we should further explore the use of nanoceria as

- An anticancer agent for the treatment of all kinds of cancer which is one of the most challenging tasks,
- A targeted drug and gene delivery agent for the treatment of various life-threatening diseases, like cancer, cardiac disease, and Alzeimer’s disease, etc.
- An anti-diabetic agent to treat diabetes mellitus and its associated organ dysfunction which is one of the significant health problems worldwide, and
- A useful scaffold in tissue regeneration field.

Based on these considerations, the use of nanoceria needs to be further explored so that it can be used as an active therapeutic agent in biomedicine.

## Data Availability

Not applicable.

## Abbreviations

BMSCs: bone marrow stromal cells
CAT: catalase
CNP: cerium oxide nanoparticle
CTAB: cetyltrimethylammonium bromide
DODAB: dimethyldioctadecylammonium bromide
DOTAP: 1,2-dioleoyloxy-3-trimethylammonium propane
HMSCs: human mesenchymal stem cells
hCAII: human carbonic anhydrase enzymeII
IDDM: insulin dependent diabetes mellitus
IDF: international diabetes federation
MSN: mesoporous silica nanoparticle
NSCLC: non-small-cell lung cancer
PPCNPs: polyethylenimine-PEGylation ceria nanoparticles
PAA: polyacrylic acid
PVP: polyvinyl pyrrolidone
ROS: reactive oxygen species
SHS: self-propagating high-temperature synthesis
SOD: superoxide dismutase
STZ: streptozotocin
